# High Free‐Volume Imidazole‐Based Ionomers for High‐Temperature Proton Exchange Membrane Fuel Cells

**DOI:** 10.1002/advs.75891

**Published:** 2026-06-01

**Authors:** Ge Chao, Hyeon Keun Cho, Chang Yeon Hyun, Shirong Li, Jong Geun Seong, So Young Lee, Nanwen Li, Young Moo Lee

**Affiliations:** ^1^ Department of Energy Engineering College of Engineering Hanyang University Seoul Republic of Korea; ^2^ Center For Hydrogen and Fuel Cells Korea Institute of Science and Technology (KIST) Seoul Republic of Korea; ^3^ Division of Energy & Environment Technology KIST School University of Science and Technology (UST) Seoul Republic of Korea; ^4^ Faculty of Materials Science and Energy Engineering Shenzhen University of Advanced Technology Shenzhen China

**Keywords:** backbone engineering, high temperature proton exchange membrane, ionomer, phosphoric acid doping, poly(aryl imidazole)

## Abstract

Excessive swelling and mechanical degradation of high‐temperature proton exchange membranes (HT‐PEMs) compromise interfacial stability and long‐term durability, although high phosphoric acid (PA) doping is required for sufficient proton conductivity. Addressing this trade‐off requires ionomers capable of sustaining efficient charge and mass transport under reduced PA contents. Herein, a series of poly(aryl imidazole) ionomers (PA4IM‐x) with comparable ion‐exchange capacities is rationally engineered through backbone modulation to introduce tailored architectures with controlled fractional free volume. By decoupling ion‐exchange capacity from skeletal structure, the intrinsic effects of backbone geometry on physicochemical properties, catalyst‐layer morphology, and electrochemical performance are systematically elucidated. Among the investigated materials, the fluorene‐based ionomer (PF4IM‐72) achieves an optimal balance between PA uptake, dimensional stability, proton conductivity, and gas permeability. This balanced transport behavior enhances catalyst utilization and interfacial kinetics, enabling an H_2_/O_2_ fuel cell to deliver a peak power density of 0.838 W cm^−^^2^ at 200°C without backpressure, even when paired with a low‐swelling HT‐PEM. These findings establish backbone engineering as an effective molecular strategy to regulate interfacial transport and advance next‐generation hydrocarbon ionomers for durable HT‐PEMFCs.

## Graphical abstract

High free‐volume imidazole‐based ionomers (PF4IM‐72) enable enhanced phosphoric acid uptake and efficient proton transport, delivering peak power densities of 0.645 W cm^−^^2^ at 160 °C and 0.838 W cm^−^^2^ at 200 °C in HT‐PEMFCs. This work highlights a promising platform for next‐generation ionomers with improved high‐temperature performance and stability.

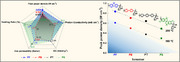

## 1 Introduction

High‐temperature proton exchange membrane fuel cells (HT‐PEMFCs) offer several intrinsic advantages over their low‐temperature counterparts, including simplified water management, enhanced tolerance to fuel impurities (e.g., CO), improved electrode kinetics, and favorable heat integration at operating temperatures typically between 120°C and 200°C [1, 2]. Among the membrane materials developed for HT‐PEMFCs, phosphoric acid (PA)‐doped polybenzimidazole (PBI) remains the most widely employed high‐temperature proton exchange membrane (HT‐PEM) due to its excellent thermal stability, chemical robustness, and ability to conduct protons under anhydrous conditions. However, the growing demand for sustainable energy technologies necessitates the development of next‐generation membrane and ionomer materials capable of delivering simultaneously high performance and long‐term durability.

In recent years, various aromatic heterocyclic polyelectrolytes bearing cationic groups—such as quaternary ammonium [3, 4, 5, 6], piperidine [7], imidazole [8, 9], pyridine [10], sulfonimide [11], and guanidinium [12] moieties—have been extensively explored as alternative materials for HT‐PEM applications. These polymers, often synthesized via superacid‐catalyzed reactions, provide tunable backbone architectures and acid–base interactions. The electrochemical performance of HT‐PEMFCs is fundamentally governed by the proton conductivity of the membrane electrolyte, making the development of highly conductive yet mechanically robust membranes essential.

For example, Yang et al. [13] introduced benzimidazole units in poly(terphenyl piperidinium) membranes (PTP‐BeIm), increasing the PA doping level to 215% and achieving a proton conductivity of 88 mS cm^−1^. Compared to PTP‐M (59% of PA uptake, ∼30 mS cm^−1^), the PTP‐BeIm membrane achieved a high peak power density (PPD) of 1 W cm^−2^ at 180°C without backpressure. However, the mechanical strength of BeIm membrane at 215%PA (3.3 MPa) was significantly lower than that of PTP‐35% with 59%PA (7.8 MPa). This example highlights a critical challenge: although high acid doping levels (ADLs) enhance proton conductivity, they substantially compromise mechanical integrity [14]. Since long‐term stable operation requires not only high conductivity but also robust mechanical strength and chemical resistance, developing HT‐PEMs with high swelling resistance against PA plasticization and strong acid anchoring capability remains imperative.

To address this conductivity‐mechanical trade‐off, considerable efforts have been devoted to structural optimization of polymer backbone through branching [10, 15], crosslinking [16], incorporation of intrinsic microporosity [8, 9], and composite membrane design [17]. For example, He et al. developed branched PTP/2%TCB membranes that increased the dry‐state tensile strength from 45.4 to 50.6 Mpa [10]. Nevertheless, after PA doping (277%), the tensile strength decreased dramatically to 6.06 MPa due to acid plasticization, even lower than that of the PA‐doped linear PTP membrane (11.3 MPa). Therefore, breaking the inherent trade‐off between proton conductivity and mechanical stability remains a central challenge in HT‐PEM design.

Beyond membrane optimization, an alternative and complementary strategy is to employ membranes with intrinsically low swelling as stable platforms while compensating for their moderate conductivity by optimizing electrode ionomers and interfacial transport. Ionomers critically influence catalyst layer performance by regulating proton transport, reactant accessibility, and catalyst utilization [4, 18, 19, 20]. Their properties are strongly determined by chemical functionality and backbone architecture. Common ionomers include PTFE [21, 22, 23, 24, 25], PBI [22, 26, 27, 28, 29], and phosphonic acid‐containing polymers [18, 30, 31, 32] and other nitrogen‐containing polymers. In our previous studies, 4‐imidazole‐based polymers employed as ionomers exhibited promising fuel cell performance, demonstrating their potential in catalyst layer engineering [33]. However, systematic backbone optimization of imidazole‐based ionomers remains largely unexplored.

Polymer backbone architecture has been shown to exert a profound influence on physicochemical properties across various electrochemical energy conversion systems. Backbone engineering has recently been intensively investigated in anion exchange membranes (AEMs), anion exchange ionomers (AEIs), and proton exchange membranes (PEMs), proton exchange ionomers (PEIs), etc. For example, Kim et al. designed a poly(fluorene) ionomer (FLN55) that minimized undesirable phenyl interaction with hydrogen oxidation catalysts, enabling an AEI with a higher PPD of ∼1.00 W cm^−^^2^ than biphenyl‐based BPN AEI (0.65 W cm^−^^2^) at 80°C with fully humidified H_2_/O_2_ (500 sccm/300 sccm) with 285 kPa backpressure [34]. Ding et al. introduced dimethylfluorenylene (DMeF) units (81 mol%) to increase free volume in PFTPA‐81 AEM, achieving higher ion conductivity (177 mS cm^−^^1^) and PPD of 1.33 W cm^−^^2^ than original PTPA AEM (0.83 W cm^−^^2^) at 80°C and fully humidified H_2_/ O_2_ (300 sccm/300 sccm) with 1.5 bar back pressure [35]. These studies underscore the critical roles of backbone rigidity, planarity, and aromatic connectivity in governing ion transport and device performance. Similarly, Zhao et al. designed an intrinsic microporosity HT‐PEM polymer (PSBI‐IM) with high fractional free volume (FFV), achieving a proton conductivity of 50.44 mS cm^−^^1^ and a PPD of 0.615 W cm^−^^2^ at 160°C with H_2_/O_2_ (400 sccm/400 sccm) without backpressure, attributed to its high BET surface area (241 m^2^ g^−^^1^) and substantial PA uptake (216.8%). It is higher than the terphenyl‐based PTP‐IM HT‐PEM of 0.54 W cm^−^^2^ [8]. In addition, our group previously developed a spirobisindane‐based poly(aryl‐co‐aryl piperidinium) AEM and AEI exhibiting an exceptional hydroxide conductivity of 208.1 mS cm^−^^1^ at 80°C and a PPD of 2.02 W cm^−^^2^, arising from the high free volume imparted by the spiro architecture [36].

**FIGURE 1:**
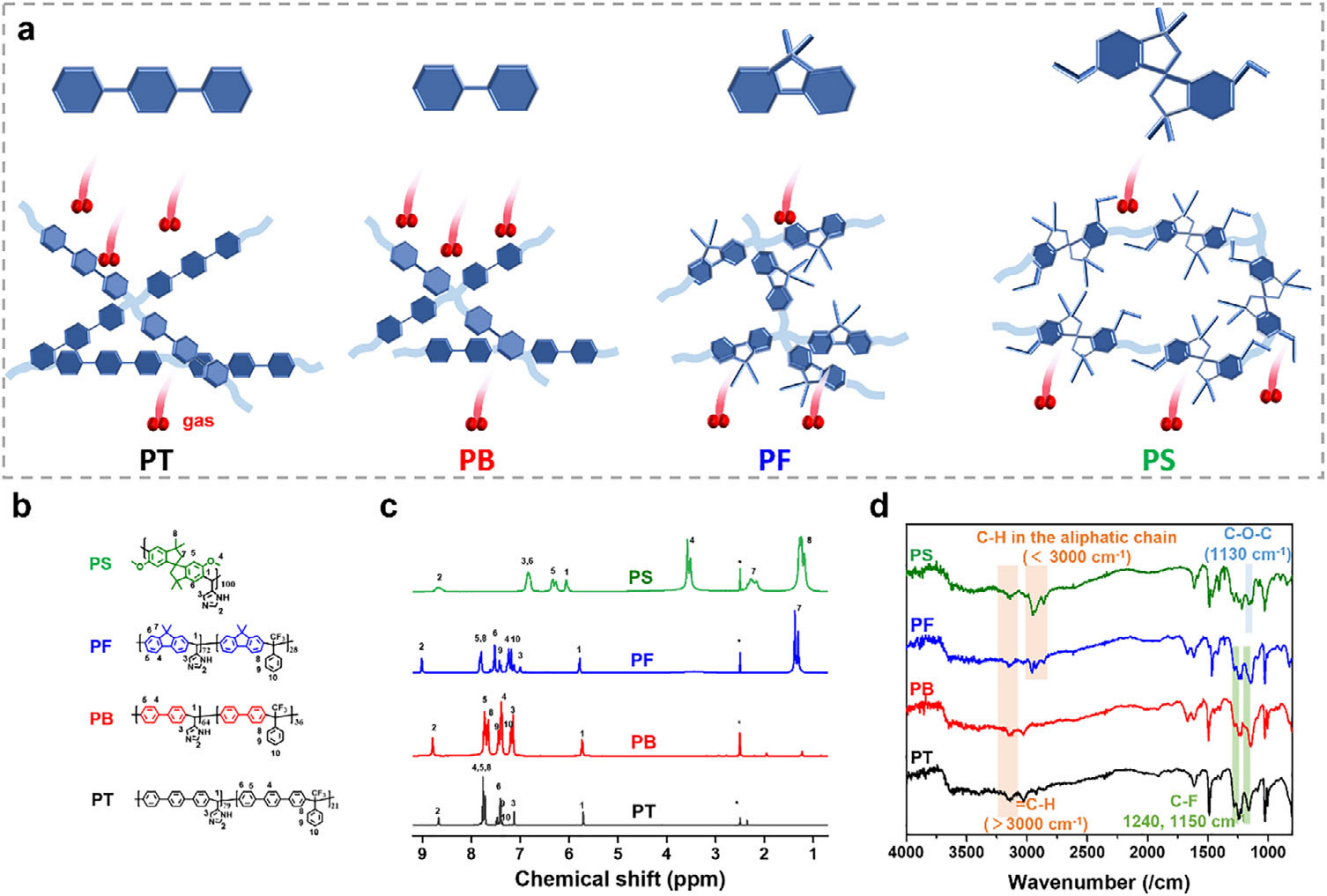
Synthesis and characterization of imidazole‐based polymers as an ionomer. (a) Schematic illustrations of aromatic monomers with distinct structural features that influence backbone extension and chain packing behavior. (b) Chemical structures of the synthesized imidazole based‐polymers (PA4IM‐x series). (c) ^1^H NMR spectrum of the polymers recorded in DMSO‐*d*_6_. (d) ATR‐FTIR spectra of the imidazole based‐polymers.

Inspired by these structure–property relationships, the present study aims to enhance HT‐PEMFC performance by rationally tailoring the backbone architecture of imidazole‐based ionomers to compensate for the limited proton conductivity of low‐swelling HT‐PEMs. Specifically, four distinct phenyl‐based backbone—biphenyl (BP), terphenyl (TP), fluorene (FL), and spirobisindane (Spiro)—are systematically investigated (Figure 1a,b and Table S1). Their effects on free fractional volume, PA uptake, proton conductivity, and mechanical properties are comprehensively evaluated. Furthermore, catalyst dispersion behavior within the catalyst layer and its correlation with electrochemical performance are examined and linked to single‐cell performance. Through this integrated investigation, we establish clear correlations between backbone structure, ionomer functionality, and device‐level performance. This work provides new insights into backbone‐regulated ionomer design and demonstrates an effective strategy for achieving high‐performance HT‐PEMFCs by integrating advanced ionomers with low‐swelling HT‐PEM systems.

## 2 Results and Discussion

### 2.1 Synthesis and Characterizations

A rationally engineered polymer backbone is essential for regulating chain conformation and intermolecular packing, which ultimately determine the physicochemical behavior of ionomers in catalyst layers. To systematically elucidate the influence of backbone architecture on ionomer properties and fuel cell performance, a series of PA4IM‐100 polymers incorporating distinct aromatic backbones were synthesized via superacid‐catalyzed polycondensation (Schemes S1 and S2). The chemical structures were confirmed by ^1^H NMR spectroscopy using DMSO‐*d_6_* (Figures S1 and S2). Here, B, T, F, and S denote biphenyl (BP), terphenyl (TP), fluorene (FL), and spirobisindane (Spiro units), respectively. Except for PS4IM‐100, the aromatic proton signals (H_4_‐H_6_) of the imidazole‐based polymers appear consistently between 7–8 ppm. In PS4IM‐100, however, the H_5_ and H_6_ phenyl protons shift upfield (6–7 ppm), which can be attributed to the strong electron‐donating effects of methoxy and aliphatic substituents. A characteristic resonance of the isopropyl unit (H_1_) is observed at 5.5–6.1 ppm across all polymers. The imidazole ring protons appear at 7.7–8.6 ppm (H_2_) and 6.7–7.1 ppm (H_3_), respectively. Additional aliphatic proton signals are observed at ∼1.3 ppm for PF4IM‐100 and at ∼3.5, 2.2, and 1.2 ppm for PS4IM‐100, confirming the successful incorporation of the designed backbone motifs.

Notably, most PA4IM‐100 polymers exhibit exceptionally high PA uptake (>1000%, Table S2), primarily due to the high density of 4‐imidazole group within the polymer framework, despite the generally low PA uptake ability reported for 4‐imidazole‐based polymer [9, 33]. To decouple excessive acid uptake from intrinsic backbone effects and to achieve comparable ion‐exchange capacities (IECs), partial substitution of imidazole units with 2,2,2‐trifluoroacetophenone (TFAP) was conducted (Scheme S3). This strategy yielded a series of PA4IM‐x copolymers—PT4IM‐79 (PT), PB4IM‐64 (PB), PF4IM‐72 (PF), and PS4IM‐100 (PS)—with controlled IECs (2.44 meq g^−1^) and varied backbone rigidity (Scheme S4). ^1^H NMR spectra of the PA4IM‐x copolymers (Figure 1c) show new aromatic proton signals corresponding to TFAP (H_8_–H_10_) in the 7–8 ppm region, confirming successful copolymer formation. Based on proton integration in the ^1^H NMR spectra, the calculated IEC values of the three copolymers are 2.56 (PT), 2.69 (PB), and 2.53 (PF) meq g^−1^ (Table 1), in good agreement with the theoretical values. Attenuated total reflectance Fourier‐transform infrared (ATR‐FTIR) spectra further verify the structures (Figure 1d). All polymers display characteristic N─H stretching (∼3400 cm^−^^1^) and aromatic ═C─H stretching (∼3024 cm^−^^1^) bands. PF and PS exhibit additional aliphatic C─H stretching bands below 3000 cm^−^^1^. The C─F stretching vibrations from TFAP appear at ∼1240 and 1150 cm^−^^1^, corroborating TFAP incorporation.

**TABLE 1:** Physical properties of PA4IM‐x polymers.

Ionomer	IEC^a)^ (meq. g^−1^)	IEC^b)^ (meq. g^−1^)	ρ (kg m^−3^)^c)^	FFV^c)^	ADL _Tit_ ^d)^	PA doping amount (%)^d)^	SR (%)^d)^	σ (mS cm^−1^)^e)^	TS (MPa)^f)^	EB (%)^f)^	TS (MPa)^g^ ^)^	EB (%)^g^ ^)^
PT	2.44	2.56	1009	5.6	3.9±0.4	173±5	20±1	27.0±3.1	12.5	50.3	96.7	33.4
PB	2.44	2.69	1017	6.3	3.9±0.4	182±12	23±1	28.6±2.8	15.8	83.0	101.2	20.2
PF	2.44	2.53	951	6.7	8.1±0.4	324±23	37±2	45.0±2.0	5.5	93.1	—	—
PS	2.44	2.44	907	8.9	12.3±0.8	695±25	62±3	48.6±5.1	4.0	226.6	—	—

^a)^ The IEC was theoretically calculated.

^b)^ The IEC value was determined by ^1^H NMR analysis.

^c)^ FFV was calculated by computer simulation, ρ denotes density.

^d)^ Basic properties were measured after PA doping at 80°C for 2 d; SR denotes swelling ratio.

^e)^ σ denotes proton conductivity measured at 80°C.

^f)^ Mechanical properties measured under PA‐doped condition; TS denotes tensile strength, and EB denotes elongation at break.

^g)^ Measurements conducted under dry conditions.

Polymers with high molecular weight are generally expected to yield more robust ionomers, which is critical for the long‐term operation of HT‐PEMFCs. The four polymers were characterized by intrinsic viscosity measurements and gel permeation chromatography (GPC) (Figure S3 and Table S1). The intrinsic viscosities were 12.20 dL g^−^^1^ (PF), 11.87 dL g^−^^1^ (PS), 9.10 dL g^−^^1^ (PB), and 4.48 dL g^−^^1^ (PT). Correspondingly, the weight‐average molecular weights (*M_w_*) follow the order: PF (508 kDa) > PS (271 kDa) > PB (111 kDa) > PT (46 kDa), consistent with the trend in intrinsic viscosity. During the superacid‐catalyzed polymerization, protonated carbonyl groups (C═O) in aldehydes and ketones are more susceptible to electrophilic substitution with aromatic units (Ar–H) bearing electron‐donating groups, such as ─OCH_3_ in the spiro unit and ─CH_3_ in the fluorene unit. Consequently, PF and PS achieve higher molecular weights within shorter reaction times due to the presence of these electron‐donating groups. In contrast, PT and PB exhibit lower molecular weights even after prolonged reaction times, as the TP and BP units lack such substituents.

Thermogravimetric analysis (TGA, Figure S4) demonstrates negligible weight loss of samples below 250°C under nitrogen, indicating sufficient thermal stability for HT‐PEMFC catalyst layer operation. Considering the similar IEC values among the polymers, the final weight loss observed at higher temperatures (∼800°C) is attributed to backbone decomposition. Polymers with rigid aromatic backbones (PT and PB) exhibit higher char yields (>60 wt.%), whereas PF and PS, incorporating dimethylfluorene and spirobisindane units, show lower residual yields (≈55 wt.% and ≈40 wt.%, respectively), indicating comparatively reduced thermal robustness. Under air, the polymers undergo thermal degradation accompanied by oxidative combustion, ultimately leading to near‐zero residue, as expected for organic materials. Importantly, negligible weight loss is still observed below 250°C, confirming that these ionomers maintain sufficient oxidative stability within the operating temperature range of HT‐PEMFC cathodes [37]. These results provide a more realistic evaluation of thermal stability under practical operating conditions. Dynamic mechanical analysis (DMA, Figure S5) reveals pronounced backbone‐dependent thermomechanical behavior. PT and PB membranes exhibit higher storage moduli and glass transition temperatures (*T_g_*) compared with PF and PS. The more rigid and planar terphenyl and biphenyl structures restrict segmental mobility more effectively, whereas the bulky fluorene and spiro architectures introduce greater free volume and reduce chain packing efficiency.

Collectively, these results confirm the successful synthesis of structurally controlled PA4IM‐x ionomers and highlight the strong influence of backbone geometry on thermal and mechanical properties.

### 2.2 Structural and Transport Characteristics

**FIGURE 2:**
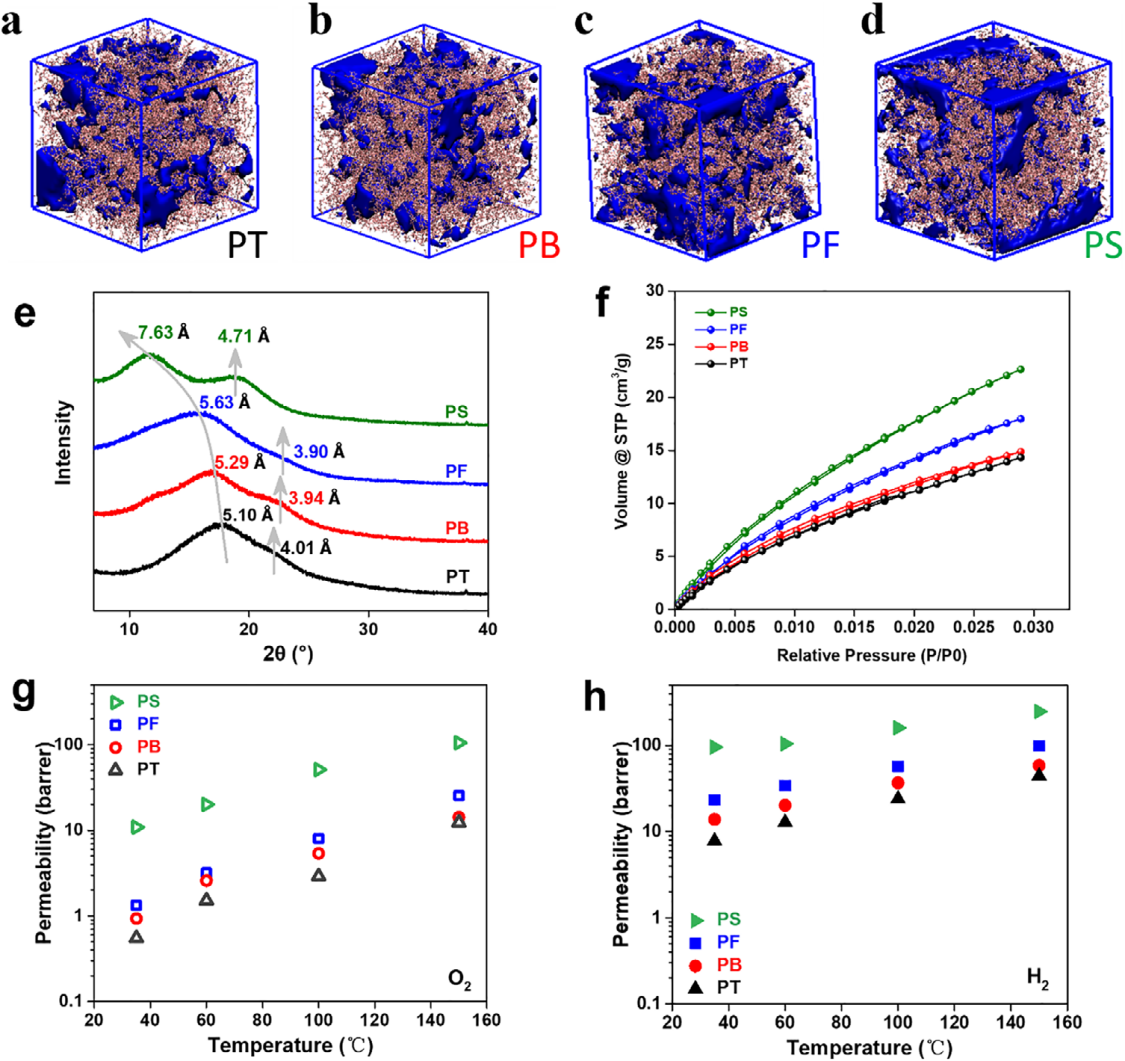
Microporous characteristics of imidazole‐based polymers and their gas transport properties. (a‐d) Three‐dimensional views of simulated amorphous cells for PA4IM‐x polymers with different backbone structures: (a) PT, (b) PB, (c) PF, and (d) PS. (e) XRD patterns of PA4IM‐x films, (f) CO_2_ adsorption isotherms at 273.15 K, (g) O_2_ permeability, and (h) H_2_ permeability of PA4IM‐x polymers in the dry state.

Incorporation of different aryl units is expected to modulate chain packing density and free volume within the polymer matrix [38, 39]. To quantify these effects, molecular simulations were performed to construct amorphous cell models (Figure 2a‐d). The calculated fractional free volume (FFV) follows the order: PS (8.9%) > PF (6.7%) > PB (6.3%) > PT (5.6%) (Table 1). The bulky spiro and fluorene structures generate larger free volume and lower packing density, consistent with their three‐dimensional steric configurations.

Wide‐angle X‐ray diffraction (WAXD, Figure 2e) reveals broad diffraction halos for all PA4IM‐x polymers, indicating predominantly amorphous structures. A low‐angle diffraction feature (2*θ* = 5.10°–7.63°) corresponds to the average interchain segment distance introduced by aryl units. The calculated d‐spacings (Bragg's law [40], λ = 1.540 Å) are: PT: 5.10 Å, PB: 5.29 Å, PF: 5.63 Å, and PS: 7.63 Å. The progressive increase in interchain spacing reflects enhanced steric hindrance from fluorene and spiro motifs, which disrupt efficient π‐conjugated stacking and lead to looser chain packing [38, 39]. A higher‐angle diffraction feature (2*θ* = 3.90°–4.01°) is attributed to *π–π* stacking interactions among arene units [41, 42]. To further probe microporosity, CO_2_ adsorption at 273.15 K was conducted (Figure 2f) [43, 44]. The CO_2_ uptake follows: PS (0.093 m^3^(STP) g^−^^1^) > PF (0.080 m^3^(STP) g^−^^1^) > PT (0.061 m^3^(STP) g^−^^1^), PB (0.054 m^3^(STP) g^−^^1^), consistent with FFV trends from simulations to be discussed later. The enhanced adsorption capacity of PS and PF confirms the presence of ultra‐microporous free volume generated by bulky backbone architectures.

Gas transport measurements were performed to evaluate the implications for catalyst layer mass transport. Pure‐gas H_2_ and O_2_ permeabilities were measured at 35°C–150°C (Figure 2g,h). For all samples, permeability increases monotonically with temperature, reflecting thermally activated diffusion. Among the series, PS exhibits the highest permeability across the entire temperature range. At 35°C, PS shows an O_2_ permeability of 10.9 Barrer, significantly higher than PF (1.3 Barrer), PB (0.9 Barrer), and PT (0.6 Barrer), so O_2_ permeability follows the order: PS > PF > PB > PT. A similar trend is observed for H_2_ permeability, following the order PS > PF > PB > PT. These results confirm that backbone architecture (BP, TP, FL, and Spiro) governs polymer chain self‐assembly and packing efficiency, thereby modulating the FFV. These results confirm that backbone‐induced increases in FFV enhance gas transport properties. The bulky spiro and fluorene units expand intermolecular spacing and reduce chain entanglement, thereby facilitating gas diffusion—an advantageous feature for catalyst layer reaction kinetics [26, 45].

Three‐dimensional molecular models (Figure S6) further illustrate backbone‐dependent conformations. The fluorene‐based PF polymer promotes partial chain folding and peripheral aggregation of ionic domains. In contrast, the terphenyl backbone in PT yields a more extended conformation, while the spiro architecture in PS forms a relatively linear yet spatially expanded chain, distributing acid‐functional groups along both sides of the backbone. These conformational differences account for the observed variations in ultra‐microporosity and chain‐packing behavior [46].

An appropriate PA doping level is essential for efficient proton transport within ionomers in the catalyst layer. Unlike bulk membrane applications, however, excessive PA uptake and severe swelling of ionomers may induce interfacial delamination and impede reactant transport within the catalyst layer. To elucidate the influence of backbone‐dependent chain packing on PA incorporation, the PA uptake, ADL, and swelling ratio of the PA4IM‐x series were systematically evaluated.

**FIGURE 3:**
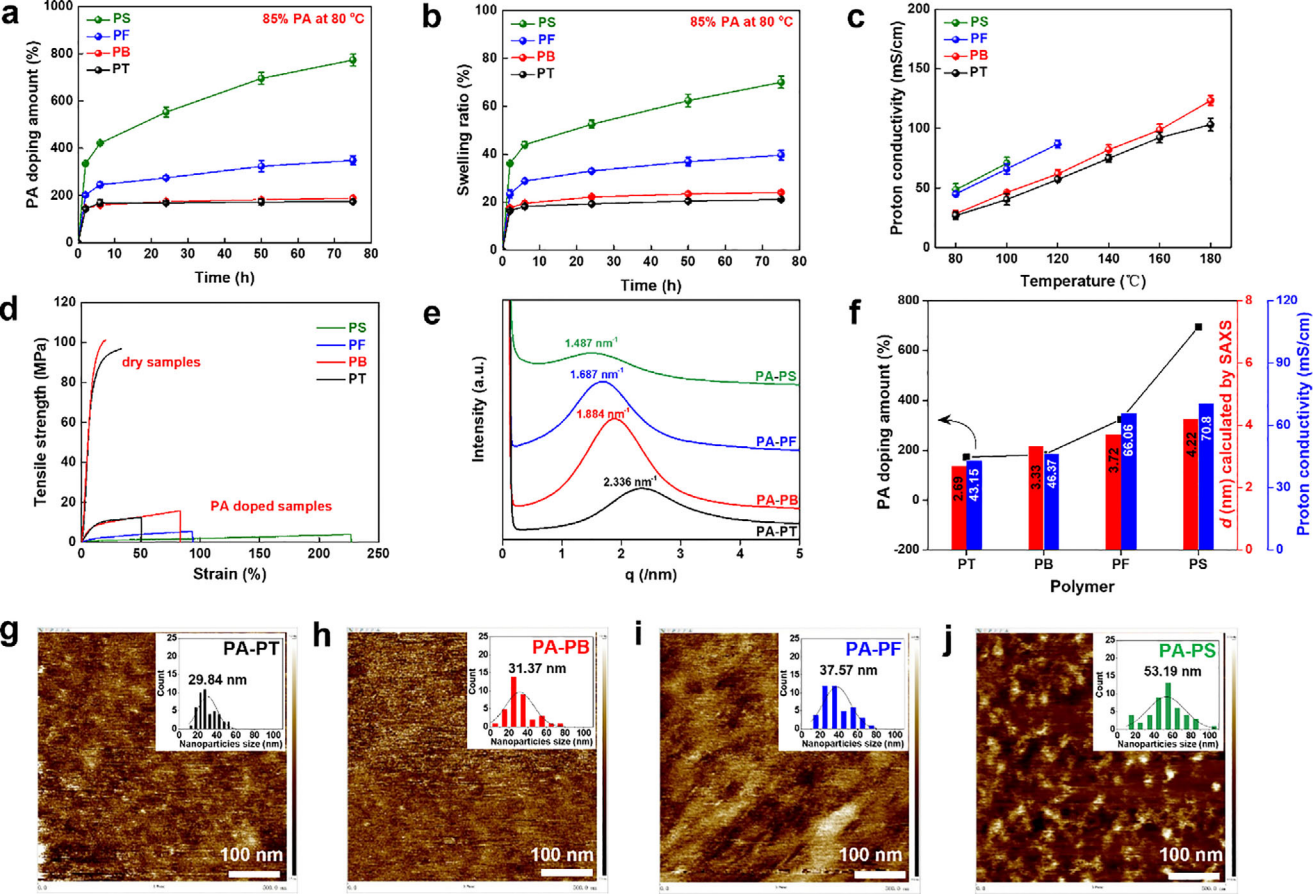
Phosphoric acid doping kinetics, morphology, and proton conductivities of PA‐doped imidazole‐based polymers. (a) Phosphoric acid (PA) doping amount and (b) swelling ratios of PA‐doped PA4IM‐x polymers. (c) Proton conductivity and (d) mechanical properties of PA‐doped PA4IM‐x films. (e) Small‐angle X‐ray scattering (SAXS) profiles of PA‐doped PA4IM‐x films. (f) Correlation between PA doping level, ionic‐cluster characteristics in PA‐doped PA4IM‐x membranes, and proton conductivity. AFM phase images of PA‐doped PA4IM‐x membranes: (g) PA‐PT, (h) PA‐PB, (i) PA‐PF, and (j) PA‐PS.

Figure 3a and Table 1 summarize the structure‐dependent PA doping behavior. With increasing doping time, the PA uptake of all polymers gradually increases, indicating a diffusion‐controlled absorption process. Despite comparable IECs, the PA4IM‐x samples exhibit markedly different PA uptake values, highlighting the dominant role of backbone architecture and chain packing density. After 2 days of doping at 80°C, PT and PB exhibit uptakes of 175% (ADL_Tit_ = 3.9) and 169% (ADL_Tit_ = 3.9), respectively. In contrast, PF reaches 275% (ADL_Tit_ = 6.3), representing an increase of nearly 50%, while PS shows a substantially higher uptake of 553% (ADL_Tit_ = 10.7), more than twice that of PT and PB. Upon prolonged doping (Table S3), PT and PB approach equilibrium after 2 days, stabilizing at 172% (ADL_Tit_ = 3.9) and 182% (ADL_Tit_ = 3.9), respectively. Conversely, PF and PS continue to absorb PA, ultimately reaching ultra‐high PA doping levels of 324% (ADL_Tit_ = 8.1) and 695% (ADL_Tit_ = 12.3), respectively. This sustained increase is attributed to the looser chain packing and higher FFV of PF and PS, which provide additional accommodation sites for PA molecules.

The swelling behavior exhibits a similar dependence (Figure 3b and Table 1). After 2 days of doping, the swelling ratio follows the order: PS (62%) > PF (37%) > PB ≈ PT (20%–23%). The incorporation of rigid and twisted spirobisindane and fluorene units disrupts efficient chain packing, thereby facilitating acid incorporation but simultaneously promoting volumetric expansion. While increased free volume enhances PA uptake, excessive swelling may compromise catalyst layer integrity and mass transport pathways, necessitating careful structural optimization.

The enhanced PA uptake is expected to improve proton conductivity. As shown in Figure 3c and Table 1, proton conductivity generally increases with PA doping level, and a near‐linear temperature dependence is observed for the PA4IM‐x polymers, consistent with thermally activated proton hopping mechanisms. However, PF and PS membranes partially dissolve or lose structural integrity at elevated temperatures due to their excessive PA uptake, preventing reliable high‐temperature conductivity measurements. The conductivity at 80°C follows the order: PS (48.6 mS cm^−1^) > PF (45.0 mS cm^−1^) > PB ≈ PT (27–29 mS cm^−1^). Notably, despite its exceptionally high PA uptake, PS does not exhibit proportionally superior proton conductivity. This deviation is attributed to excessive swelling, which disrupts continuous proton transport pathways and dilutes effective acid–base interactions. The PF polymer exhibits a significantly enhanced proton conductivity due to its optimized PA uptake and swelling, exceeding that of conventional mPBI (38 mS cm^−1^ at 80°C) [44]. Therefore, within the PA4IM‐x series, the improved proton conductivity can be primarily ascribed to backbone‐enabled PA incorporation, while excessive free volume introduces diminishing returns due to structural destabilization.

Mechanical properties before and after PA doping further illustrate the conductivity–stability trade‐off (Figure 3d and Table 1). Prior to doping, PT and PB membranes exhibit high tensile strengths of 96.7 and 101.2 MPa, respectively, with elongations at break of 20%–33%. In contrast, PF and PS membranes are brittle in the dry state and unsuitable for tensile testing, reflecting the reduced packing efficiency and ladder‐like constraints introduced by fluorene and spiro linkages [44, 47]. Their characteristic chain architectures are further elucidated by molecular modeling (Figure S6). Upon PA doping, the plasticizing effect of PA significantly reduces tensile strength while increasing elongation at break. As the PA uptake increases from 172% to 695%, tensile strength decreases from ∼16 to ∼4 MPa, whereas elongation increases from ∼50% to ∼220%. Despite this reduction in strength, the PA‐doped ionomer retain sufficient flexibility and structural integrity for catalyst‐layer applications in HT‐PEMFCs. These results indicate that imidazole‐based polymers, when employed as catalyst‐layer ionomers, provide adequate mechanical integrity to maintain structural stability, ensure effective catalyst–ionomer contact, and sustain robust interfacial adhesion under operating conditions.

Overall, these results demonstrate that backbone architecture, rather than molecular weight, governs PA uptake, swelling behavior, proton conductivity, and mechanical stability in a highly interdependent manner, as no clear correlation between molecular weight and these fundamental properties is observed. Fluorene‐ and spiro‐based backbones maximize acid incorporation and conductivity, but at the expense of dimensional and mechanical stability, whereas biphenyl and terphenyl units provide a more balanced combination of conductivity and robustness. This backbone‐dependent trade‐off is critical for rational ionomer design in HT‐PEMFC catalyst layers.

### 2.3 Morphology

Morphology control represents another critical factor governing proton transport. As shown in Figure 3e, all PA‐doped polymers in film form display broad scattering peaks in the q range of 1.4–2.4 nm^−^^1^, commonly referred to as ionomer peaks. The presence of these ionomer peaks indicates that the formation of microphase‐separated ionic domains and interconnected proton‐conducting channels, consistent with previous reports [48, 49, 50]. The characteristic ionic domain spacing was estimated using Bragg's equation (d = 2π/q), yielding values in the range of 2.6–4.3 nm. Among the samples, the PS doped with PA (PA‐PS) membrane exhibits the largest d‐spacing (4.22 nm) and the most pronounced ionic aggregation. This behavior arises from the highly twisted spiro‐based backbone structure, which increases chain mobility and free volume, thereby facilitating ion clustering and channel interconnectivity. Similarly, the PA‐PF membrane shows enlarged and well‐connected ionic domains (3.72 nm), attributed to the bulky fluorene unit. In contrast, PA‐PB (3.33 nm) and PA‐PT (2.69 nm) display smaller ionic cluster sizes, reflecting their more planar and tightly packed backbones. These results suggest that backbone rigidity, steric hindrance, and hydrophobic character collectively regulate ionic aggregation and domain spacing [48]. Consequently, distinct microphase‐separated morphologies are established across the PA‐PA4IM‐x series, which directly influence proton transport behavior (Figure 3f).

Atomic force microscopy (AFM) further visualizes the microphase separation (Figure 3g–j) of PA‐doped samples. In the phase images, dark and bright regions correspond to acidophilic and acidophobic domains, respectively. Owing to their differing degrees of ionic aggregation, the PA‐PA4IM‐x membranes exhibit distinct acidophilic‐domain morphologies. PA‐PS shows the highest degree of phase aggregation, with a characteristic acidophilic domain size of 53.19 nm. The domain size decreases progressively for PA‐PF (37.57 nm), PA‐PB (31.37 nm), and PA‐PT (29.84 nm). Importantly, all PA4IM‐x polymers contain identical concentrations of 4‐imidazole units; therefore, the observed morphological differences originate solely from variations in backbone architecture. The results confirm that spiro‐ and fluorene‐based backbones promote the formation of larger PA‐rich domains and more continuous ion‐transport channels, whereas biphenyl and terphenyl units favor more compact microphase structures.

### 2.4 Ionomer Functionality in Catalyst Layer

**FIGURE 4:**
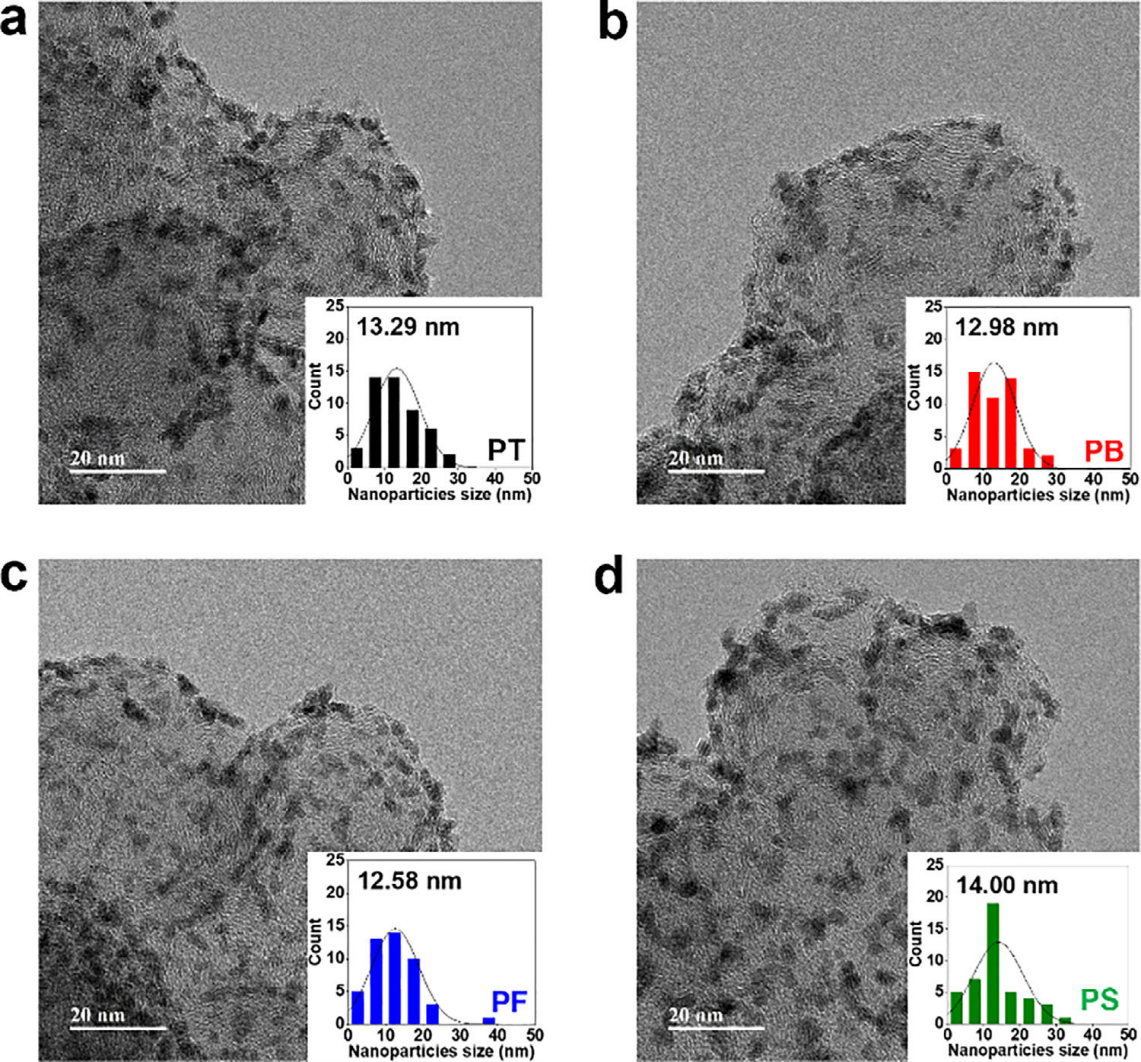
Transmission electron microscopy (TEM) images of catalyst layers prepared from different ionomers: (a) PT, (b) PB, (c) PF, and (d) PS.

To evaluate the role of backbone structure in catalyst‐layer formation, the micromorphology of Pt/C catalysts prepared with different PA4IM‐x ionomers was examined by high‐resolution transmission electron microscopy (HR‐TEM) (Figure 4). All Pt/C@PA4IM‐x catalyst layers exhibit well‐dispersed Pt nanoparticles with average particle sizes predominantly in the range of 12–14 nm, indicating effective dispersion of the catalyst with ionomers. Minor differences in aggregation behavior are observed, which can be attributed to variations in ionomer molecular architecture and interfacial interactions. Moreover, no clear correlation between molecular weight and catalyst aggregation is identified. These observations demonstrate that ionomer backbone structure plays a decisive role in regulating catalyst ink dispersion and nanoparticle distribution, thereby influencing catalyst‐layer microstructure. The steric configuration and polarity of the ionomer affect polymer adsorption onto Pt/C surfaces, particle–particle interactions, and ink rheology, ultimately governing catalyst utilization.

Scanning electron microscopy (SEM) images of the corresponding catalyst layers (Figure S7) reveal uniformly distributed microporous structures without large agglomerates. Well‐developed void networks of varying sizes are observed in all samples, confirming homogeneous Pt/C dispersion within the catalyst layer. It is well established that ionomer properties critically influence ink dispersion by modulating surface charge, polymer bridging behavior, and interfacial interactions among Pt/C particles, solvent molecules, and ionomer chains [51]. In this context, backbone‐induced variations in chain flexibility, free volume, and acid affinity are expected to influence catalyst‐layer porosity, reactant accessibility, and proton transport continuity. Thus, the molecular design of PA4IM‐x ionomers not only governs intrinsic proton conductivity but also plays a pivotal role in constructing optimized catalyst‐layer architectures for enhanced HT‐PEMFC performance.

### 2.5 Fuel Cell Performance

**FIGURE 5:**
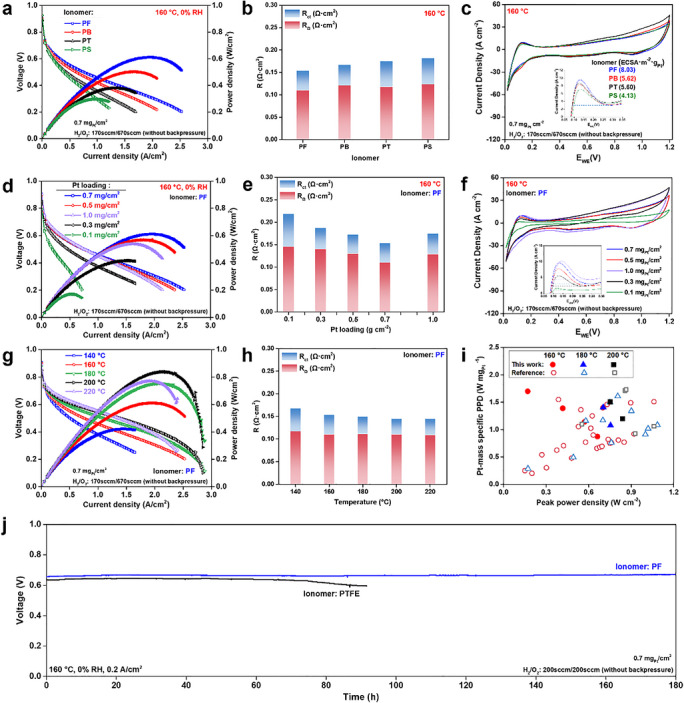
Fuel cell performance of MEAs based on PA4IM‐x ionomers and PA‐doped PT4IM membrane. (a,d,g) Polarization curves of MEAs based on PA4IM‐x ionomers and a PT4IM/PA membrane (55 ± 5 µm): (a) different PA4IM‐x ionomers at 160°C; (d) PF ionomer at 160°C (Pt loading: 0.1–1.0 mg Pt cm^−^^2^); (g) PF ionomer at 140°C–220°C (Pt loading: 0.7 mg Pt cm^−^^2^). (b,e,h) Corresponding charge‐transfer resistance (R_ct_) and ohmic resistance (R_Ω_) under identical conditions. (c,f) Cyclic voltammetry (CV) curves recorded under H_2_/N_2_ at 160°C (Pt loading: 0.1–1.0 mg Pt cm^−^^2^). (i) Literature comparison of HT‐PEMFC performance using imidazole‐based membranes (data compiled from Tables S4 and S5). (j) Single‐cell durability at 160°C under a constant current density of 200 mA cm^−^^2^.

To access the practical applicability of the PA4IM‐x polymer family as ionomers in catalyst layers, membrane–electrode assemblies (MEAs) were fabricated using PA‐PT4IM membranes (thickness: 55 ± 5 µm) and evaluated in HT‐PEMFC single cells. Performance was measured under H_2_/O_2_ operation with flow rates of 170 and 670 sccm, respectively. Polarization curves of the PA4IM‐x ionomer were recorded in anhydrous conditions without backpressure (Figure 5a and Figures S8 and S9). At 160°C and 0% RH, all cells exhibited open‐circuit voltages (OCVs) exceeding 0.9 V (Table 2), indicating good membrane integrity and minimal gas crossover. Despite identical membrane conditions, the overall fuel cell performance strongly depends on the type of ionomer employed in the catalyst layer, highlighting the decisive role of ionomer backbone architecture. The peak power density (PPD) follows the order: PF > PB > PT > PS. Notably, when PF was used as the ionomer, the fuel cell delivered a PPD of 0.611 W cm^−^^2^ at a current density of 0.323 A cm^−^^2^. This value is approximately 0.109 W/cm^2^ higher than that achieved with PB (0.502 W cm^−^^2^) and 0.232 W/cm^2^ higher than that obtained with PT (0.379 W cm^−^^2^). In contrast, PS exhibited the lowest performance despite its highest intrinsic proton conductivity. This trend highlights a critical structure–performance interplay. Although the PS polymer possesses the highest fractional free volume and PA uptake, its excessive swelling and enlarged ionic domains likely compromise catalyst‐layer stability and interfacial proton transport continuity. Conversely, high molecular weight PF achieves a more balanced combination of moderate free volume, well‐connected ionic channels, controlled swelling, and favorable catalyst dispersion, resulting in superior electrochemical performance. These results demonstrate that optimal HT‐PEMFCs performance does not arise from maximizing proton conductivity alone, but rather from achieving a synergistic balance among proton transport, gas permeability, dimensional stability, and catalyst‐layer microstructure. Backbone engineering of imidazole‐based ionomers, therefore, represents an effective strategy for tuning this multi‐parameter optimization.

**TABLE 2:** Fuel cell performance of single cell with various ionomers and PA‐doped PT4IM membrane measured at 160°C and 0% RH.

Ionomer	OCV(V)	PPD (W cm^−2^)^a)^	Pt‐mass specific PPD (W mg_Pt_^−1^)^b)^	R_Ω_ (Ω·cm^2^)	R_CT_ (Ω·cm^2^)	ECSA (m^2^ g_Pt_^−1^)
PF	0.91	0.611	0.873	0.110	0.0437	8.03
PB	0.90	0.502	0.727	0.121	0.0457	5.62
PT	0.90	0.379	0.541	0.118	0.0575	5.60
PS	0.92	0.297	0.424	0.123	0.0586	4.13

^a)^ Peak power density @ 160°C and 0% RH.

^b)^ Pt‐mass specific PPD (W mg_Pt_^−1^) = peak power density (W cm^−2^) / Pt loading of the cathode (mg_Pt_ cm^−2^).

Electrochemical impedance spectroscopy (EIS) analysis and internal resistance measurements (Figure 5b and Figures S8 and S9 and Table 2) further elucidates the origin of the performance differences. The PF‐based MEA exhibits the lowest ohmic resistance of 0.110 Ω·cm^2^ and charge transfer resistance (R_CT_ = 0.0437 Ω·cm^2^), accounting for its superior electrochemical performance. The reduced ohmic resistance reflects efficient proton conduction across both the membrane and catalyst layer, while the low R_CT_ indicates enhanced electrode kinetics. These improvements arise from the synergistic combination of moderate free volume, sufficient fuel permeability, well‐connected proton transport pathways within the catalyst layer—consistent with its highest observed PPD.

In contrast, despite its highly loose chain packing and elevated proton conductivity, PS delivers the lowest PPD (0.297 W cm^−2^ at 0.311 A cm^−2^). This inferior performance is attributed to excessive PA uptake, which induces electrode flooding and obstructs gas diffusion, thereby severely limiting mass transport within the catalyst layer. Thus, excessive free volume and swelling lead to diminishing electrochemical returns due to transport limitations. Except for PS ionomer, the current densities of the fuel cells in the high‐voltage (kinetic‐controlled) region follow the same trend as fuel permeability and proton‐transport capability of the corresponding ionomers, confirming that balanced transport properties are essential for optimizing oxygen reduction reaction (ORR) kinetics.

Platinum catalyst utilization in the presence of different ionomers was evaluated by cyclic voltammetry (CV) through hydrogen desorption analysis (Figure 5c and Table 2). Among the samples, the PF ionomer achieves the highest electrochemically active surface area (ECSA) of approximately 8.03 m^2^ g^−1^; significantly exceeding those of PB (5.62 m^2^ g^−1^) and PT (5.60 m^2^ g^−1^m^2^ g^−1^). The enhanced ECSA indicates improved catalyst accessibility and dispersion, which can be attributed to the optimized backbone architecture that provides sufficient FFV without inducing excessive swelling. These results collectively demonstrate that introducing aryl backbones with controlled steric expansion enhances proton transport, fuel permeability, and catalyst utilization in a coordinated manner. Importantly, optimal HT‐PEMFC performance arises from a balanced interplay among ionic conductivity, gas diffusion, dimensional stability, and interfacial kinetics, rather than from maximizing any single parameter.

As discussed above, the fuel cell employing the PF ionomer delivers the highest PPD among the PA4IM‐x series. To further optimize HT‐PEMFC performance, the Pt catalyst loading in the catalyst layer was systematically varied from 0.1 to 1.0 mg_Pt_ cm^−2^ at 160°C (Figure 5d and Figure S10). When the Pt loading increased from 0.1 to 0.7 mg_Pt_ cm^−2^, the PPD increased markedly from 0.170 to 0.611 W cm^−2^. At this optimal loading (0.7 mg_Pt_ cm^−^^2^), the cell exhibits the lowest ohmic resistance (R_Ω_ = 0.110 Ω cm^2^), a low charge‐transfer resistance (R_ct_ = 0.0437 Ω cm^2^), and a relatively high electrochemically active surface area (ECSA = 8.03 m^2^ g_Pt_^−1^), indicating efficient catalyst utilization and well‐balanced mass and charge transport within the catalyst layer (Figure 5e,f and Figure S10). However, further increasing the Pt loading to 1.0 mg_Pt_ cm^−2^ results in performance deterioration, with the PPD decreasing to 0.539 W cm^−^^2^. This decline is primarily attributed to the increased catalyst layer thickness (Figure S11), which aggravates mass‐transport limitations. Consequently, both ohmic resistance (R_Ω_ = 0.129 Ω cm^2^) and charge‐transfer resistance (R_ct_ = 0.0457 Ω cm^2^) increase, while the ECSA decreases significantly to 4.65 mg_Pt_ cm^−2^, reflecting reduced catalyst accessibility and less efficient utilization.

The FC performance is strongly dependent on temperature due to the enhanced electrochemical reaction kinetics and proton conductivity [52, 53]. Polarization curves were recorded over a temperature range of 140°C–220°C under anhydrous conditions without backpressure (Figure 5g and Figures S8 and S9). As the temperature increased from 140°C to 200°C, the PPD of the PF ionomer increased markedly from 0.442 to 0.838 W cm^−2^. Correspondingly, R_Ω_ decreased slightly by 6% (from 0.117 to 0.110 Ω cm^2^), whereas R_ct_ decreased more significantly by 32% (from 0.0502 to 0.0340 Ω cm^2^) in Figure 5h. This indicates that the temperature dependence of R_ct_ is dominated by accelerated electrode reaction kinetics, particularly in the low‐current‐density (kinetic) region, while R_Ω_ is less sensitive to temperature under these conditions [54]. As summarized in Figure 5i and Tables S4 and S5, a high Pt mass–specific power density of 1.14 to 1.70 W mg_Pt_^−1^ at 160°C is achieved for fuel cells employing the PF ionomer. These values are highly competitive with those reported for state‐of‐the‐art MEAs (typically 0.20–1.70 W mg_Pt_^−1^), including high‐performance imidazole‐based PEMs, PA‐doped PBI membranes, and piperidinium‐based PEMs, as well as advanced ionomer systems, such as PTFE‐, PBI‐, as well as phosphorylated polymer‐based materials. This expanded comparison provides a more comprehensive benchmark and clearly demonstrates the competitive performance and practical potential of the PF ionomer in high‐temperature fuel cell applications.

The performance advantage of the PF ionomer is further corroborated by its outstanding operational stability, maintaining a constant current density of 200 mA cm^−^^2^ for over 170 h at 160°C. This durability surpasses that of conventional PTFE‐based ionomers in HT‐PEMFCs (Figure 5j). The improved stability of the PF‐based MEA originates from its stronger PA affinity compared to the PTFE ionomer [26], which effectively suppresses PA loss and maintains stable catalyst‐layer functionality during operation. Collectively, these results demonstrate that an optimized ionomer design effectively mitigates the adverse effects associated with low PA doping levels in HT‐PEM. By enabling high catalyst utilization and efficient mass transport at reduced PA contents, the tailored ionomer suppresses excessive swelling while maintaining favorable proton conduction pathways, thereby enhancing both operational stability and overall device performance.

## 3 Conclusions

In this work, a series of poly(aryl imidazole)–based ionomers with distinct backbone architectures was successfully developed to systematically elucidate structure–property–performance relationships in HT‐PEMFCs. Imidazole‐based polymers (PA4IM‐x, where x denotes the imidazole ratio, and A represents different aromatic backbones, including biphenyl (B), terphenyl (T), dimethylfluorene (F), and spirobisindane (S)) were synthesized via superacid‐catalyzed Friedel–Crafts‐type polycondensation. All ionomers were designed with comparable ion‐exchange capacities (IEC ≈ 2.44 meq g^−1^), enabling an unbiased assessment of backbone structural effects. By employing ionomers with identical IECs but distinct skeletal architectures, we systematically clarified their influences on intrinsic physicochemical properties, ink microstructure, catalyst‐layer morphology, and electrode‐electrolyte interfacial characteristics. Comprehensive electrochemical evaluation revealed that backbone architecture critically governs phosphoric acid uptake, swelling behavior, free fractional volume, and transport characteristics within the catalyst layer. Among the investigated materials, the fluorene‐based ionomer (PF) exhibited an optimal balance between phosphoric acid uptake, dimensional stability, and microporosity. This balanced architecture promoted improved interfacial compatibility and facilitated efficient proton and gas transport within the catalyst layer. Notably, even when paired with a PT4IM‐based membrane possessing relatively low proton conductivity, the PF ionomer enabled outstanding fuel cell performance, achieving peak power density of 0.611 W cm^−^^2^ at 160°C and 0.838 W cm^−^^2^ at 200°C under anhydrous conditions without backpressure with 0.7 mg_Pt_ cm^−2^ Pt loading. These findings demonstrate that rational backbone engineering of imidazole‐based ionomers can effectively compensate for intrinsic membrane limitations by enhancing catalyst‐layer functionality. Overall, this study underscores the pivotal role of ionomer backbone architecture in dictating electrochemical kinetics and mass transport in HT‐PEMFCs, and provides a clear molecular design strategy for the development of next‐generation, high‐performance imidazole‐based ionomers.

## 4 Experimental Section

### 4.1 Materials

Bisphenol A, p‐terphenyl (TP, >99.5%), biphenyl (BP, >99.5%), 9,9‐dimethylfluorene (DF, >99%), trifluoromethanesulfonic acid (TFSA), trifluoro acetophenone (TFAP, >98%), and 1H‐imidazole‐4‐carbaldehyde (4‐IM) were purchased from TCI Development Co., Ltd (Tokyo, Japan). Methanesulfonic acid (MSA), potassium carbonate (K_2_CO_3_), was purchased from Sigma–Aldrich Chemical (Milwaukee, WI, USA). Trifluoroacetic acid (TFA), bisphenol A, and other chemicals were purchased from Daejung Chemicals & Metals (Siheung‐si, Gyeonggi‐do, Korea). All chemicals were used without further purification unless otherwise stated

### 4.2 Synthesis of Monomers

Synthesis of TTSBD: 3,3,3′,3′‐Tetramethyl 2,2′,3,3′‐tetrahydro‐1,1′‐spirobisindane−6,6′‐diol (TTSBD) was synthesized according to our previous reports [36]. Briefly, bisphenol A (200 g) and methanesulfonic acid (20 mL) were added to a 250 mL round‐bottom flask equipped with magnetic stirring. The mixture was heated to 135°C and maintained for 4 h. During the reaction, the solution color gradually changed from wine red to deep red. The reaction mixture was then poured into 800 mL of deionized water to precipitate the crude product. The solid was collected and purified by recrystallisation from an ethanol/water mixture (40/60, w/w and 30/70, w/w), yielding a white needle‐shaped product. The purified TTSBD was dried in a convection oven at 80°C for 24 h.

Synthesis of Demethylated TTSBD (Dm‐TTSBD): TTSBD (20.0 g, 65 mmol) was dissolved in 200 mL of N,N‐dimethylformamide (DMF). Potassium carbonate (33.3 g, 260 mmol) and iodomethane (55.4 g, 390 mmol) were added, and the reaction was carried out at room temperature under dark conditions for 24 h. The reaction mixture was slowly poured into ice water with vigorous stirring to precipitate the product. The resulting white solid was washed several times with deionized water and dried in a vacuum oven at 70°C for 24 h.

### 4.3 Synthesis of PA4IM‐x Polymers

All PA4IM‐x polymers were synthesized via superacid‐catalyzed Friedel–Crafts‐type polycondensation, following our previous reports. In this notation, “A” denotes the aryl backbone (T = terphenyl, B = biphenyl, F = dimethyl fluorene, S = spirobisindane), and x represents the molar ratio of the imidazole‐containing monomer.

Representative Procedure (PT4IM): p‐Terphenyl (TP, 10 mmol, 2.301 g) and 1H‐imidazole‐4‐carbaldehyde (4‐IM, 11 mmol, 1.056 g) were dissolved in dichloromethane (DCM, 15 mL) in a 100 mL three‐necked flask under nitrogen. Methanesulfonic acid (MSA, 6 mL) and trifluoromethanesulfonic acid (TFSA, 6 mL) were added dropwise at sub‐zero temperature using an ice bath. After complete addition, the reaction mixture was stirred for 2 h, during which the viscosity increased markedly. The viscous solution was then precipitated into deionized water, and the resulting polymer was washed three times with water at room temperature. The product was collected by filtration and dried at 80°C.

Other PA4IM‐x polymers were synthesized using similar procedures with adjusted monomer ratios and acid compositions. Detailed polymerization conditions are summarized in Table S1, and all polymers were obtained in yields ranging from 82%–95%.

### 4.4 Membrane Preparation

PA4IM‐x polymers were dissolved in dimethyl sulfoxide (DMSO) to prepare 6 wt.% polymer solutions. The solutions were filtered through a PTFE membrane filter (pore size 1 µm) to remove impurities. The filtered solutions were cast onto clean glass plates and dried in a vacuum oven at 80°C for 24 h. After solvent removal, transparent membranes were obtained and carefully peeled from the glass substrates.

### 4.5 Characterization

#### 4.5.1 ^1^H Nuclear Magnetic Resonance (^1^H NMR)

The chemical structures of the synthesized polymers were confirmed by ^1^H NMR spectroscopy (VNMRS 600 MHz, Varian, CA, USA) using DMSO‐*d6* as the solvent. Chemical shifts were referenced to the residual solvent peak (2.5 ppm).

#### 4.5.2 Intrinsic Viscosity

The intrinsic viscosity ([ŋ]) of PA4IM‐x polymers was measured using a Schott Viscometry System (AVS 370, Germany) equipped with a Ubbelohde viscometer (SI Analytics, Type 530 13, Capillary No. Ic, K = 0.03) and an automatic piston burette (TITRONIC universal). Measurements were conducted in DMSO solution at 25°C. A stock polymer solution (3 mg mL^−1^) was prepared and automatically diluted to concentrations of 3.0, 2.5, 2.0, 1.5, and 1.0 mg mL^−1^. Efflux times were recorded for each concentration. Intrinsic viscosity was calculated according to the following equation.

(1) $$\left[\eta \right]=\lim_{c\to 0}\left(\frac{\frac{t}{t_{0}}-1}{c}\right)$$

where t_0_ denotes the efflux time of pure DMSO, and c and t are the polymer concentration and the efflux time of the solution, respectively.

#### 4.5.3 Gel Permeation Chromatography (GPC)

The molecular weight and molecular weight distribution of the polymers were determined using a gel permeation chromatograph (GPC 50, Agilent Technologies, Santa Clara, CA, USA) equipped with a refractive index detector. DMSO containing 0.01 M LiBr was used as the eluent. The system was calibrated with polystyrene standards to obtain the number‐average molecular weight (Mn), weight‐average molecular weight (Mw), and polymer dispersity index (PDI), defined as Mw/Mn.

#### 4.5.4 Mechanical Properties

Tensile properties of the membranes in the dry state or PA‐doping state were measured using a universal testing machine (AGS‐J 500 N, Shimadzu, Japan). Membranes were cut into dumbbell shapes (2 × 10 mm^2^ gauge area) and tested at 1 mm min^−^^1^ under ambient conditions. The tensile strength and elongation at the break of the membranes were measured under ambient conditions at a stretching rate of 1 mm/min. Dynamic mechanical analysis (DMA, a Discovery DMA 850, TA Instruments, New Castle, DE, USA) was performed under N_2_ with a heating rate of 5°C min^−^^1^. The storage modulus, loss modulus, and tan delta of the membranes were evaluated by a preload force of 0.01 N and a force track of 125%.

#### 4.5.5 Thermogravimetric Analysis (TGA)

Thermal stability of the membranes was measured using a thermogravimetric analyzer (TGA, Q500, New Castle, DE, USA) under a N_2_ atmosphere (50 mL/min) from 50°C to 800°C at a 10°C min^−1^ ramping rate.

#### 4.5.6 Wide‐Angle X‐Ray Diffraction (WAXD)

Wide‐angle X‐ray diffraction (WAXD) patterns were recorded on a D8 Discover diffractometer (Bruker, Karlsruhe, Germany) equipped with a GADDS detector, over the 2θ range of 5–50° at a scanning rate of 2° min^−^^1^. Cu Kα radiation (λ = 1.5418 Å) was used. The d‐spacing values were calculated according to Bragg's law.

#### 4.5.7 Atomic Force Microscopy (AFM)

By using an atomic force microscopy equipment (AFM, Bruker Co., Billerica, MA, USA) with a silicon‐based n‐type cantilever in tapping mode to get the morphology images of membranes with a scanning size of 500 nm × 500 nm and a scanning frequency of about 0.9 Hz.

#### 4.5.8 Small‐Angle X‐Ray Scattering (SAXS)

Small‐angle X‐ray scattering (SAXS) measurements of the membranes were carried out using a SAXSess mc^2^ instrument (Anton Paar, Graz, Austria) operated at 40 kV and 50 mA with Cu Kα radiation (λ = 1.54 Å).

#### 4.5.9 Electron Microscopy Analyses

The morphology of the catalyst layers before the electrochemical tests was examined by transmission electron microscopy (TEM) using a JEM‐ARM200 microscope (JEOL, Tokyo, Japan) operated at an accelerating voltage of 200 kV.

#### 4.5.10 CO_2_ Adsorption Isotherms

CO_2_ adsorption isotherms of samples were measured using an Autosorb‐iQ surface area and porosity analyzer (Quantachrome Instruments, Boynton Beach, FL, USA).

### 4.6 Phosphoric Acid Uptake and Swelling

Dry membranes (1.0 × 4.0 cm^2^) were immersed in 85% H_3_PO_4_ solution at 80°C for varying durations. The phosphoric acid (PA) uptake and dimensional changes were recorded. PA uptake and swelling were calculated from the following equation:

(2) $$PA\;\;uptake\;amount\;\left(\%\right)=\frac{W_{wet}-W_{dry}}{W_{dry}}\times 100\%$$

(3) $$Swelling\;\left(\%\right)=\frac{l_{wet}-l_{dry}}{l_{dry}}\times 100\%$$

where W and *l* denote the weight and length of dry and PA‐doped membranes, respectively.

The PA doping level per repeat unit (ADL) was determined by acid‐base titration using 0.10 M NaOH solution and phenolphthalein as an indicator. After neutralization, membranes were thoroughly washed with deionized water, dried in a vacuum oven at 120°C for 12 h, and weighed. ADL was calculated based on the following equation:

(4) $$ADL=\frac{V_{NaOH}\times C_{NaOH}\times M_{w}}{Equiv_{mol}\times W_{dry}}$$

where used *V*_NaOH_ (V) is the volume of NaOH used in acid‐base titration, C_NaOH_ (mol/L) is the molar concentration of NaOH, and Equiv_mol_ represents the equivalent mole of titrant for PA (in this case Equiv_mol_ = 3), *Mw* (g/mol) is the molecular weight of the polymer repeat unit, *W*_dry_ (g) is the dry polymer weight. Each sample was tested three times, and the average value was calculated.

### 4.7 Proton Conductivity

Proton conductivity (σ) of PA‐doped membranes was conducted from 100 to 180°C using a four‐probe alternating current (AC) impedance analyzer (VSP and VMP3 Booster, Bio‐Logic SAS, Grenoble, France) in the frequency range from 0.1 to 100 kHz. Specific operations are as follows: Membranes were fixed in a two‐electrode cell without extra humidification, then the cell was heated and held at each temperature for 10 min to measure the proton conductivity. The anhydrous conductivity (σ) in mS/cm was calculated from:

(5) $$\sigma =\frac{d}{LWR}$$

where *d* (cm) is the distance between the two electrodes, *R* (mΩ) is the resistance of the PA‐doped membrane, *L* (cm) is the thickness of the PA‐doped membrane, and *W* (cm) is the width of the PA‐doped membrane.

### 4.8 Fractional Free Volume (FFV)

Fractional free volume (FFV) was calculated based on atomistic molecular dynamics (MD) simulations. All simulations were performed using the GROMACS (version 2022) package with the AMBER force field. All of PA4IM‐x polymer models were constructed by randomly packing 10 polymer chains, each consisting of 100 repeating units, into a cubic simulation box with an initial size of approximately 300 Å. Energy minimization was first carried out using the conjugate gradient algorithm with a maximum step size of 0.01 Å and a force convergence criterion of 100 kJ mol^−^^1^ nm^−^^1^. Subsequently, the systems were equilibrated under the NPT ensemble at 298 K for 30 ns. Temperature was controlled using the V‐rescale thermostat with a relaxation time of 0.1 ps, and pressure was regulated using the Berendsen barostat. A cutoff distance of 12 Å was applied for Lennard–Jones interactions. Long‐range electrostatic interactions were treated using the particle‐mesh Ewald (PME) method with a real‐space cutoff of 12 Å. All MD simulations were performed at 298 K with a time step of 2 fs, and the equations of motion were integrated using the leapfrog algorithm. The FFV values were calculated geometrically from the equilibrated structures using a probe sphere with a radius of 0.14 nm.

### 4.9 Electrode and MEA Fabrication

#### 4.9.1 Gas Diffusion Electrodes

Gas diffusion electrodes (GDEs) were prepared by using the catalyst‐coated substrate (CCS) method. The catalyst ink was prepared by dispersing the catalyst Pt/C and ionomer materials in organic solvents. Specifically, Pt/C (Hispec 4000, 40 wt.% Pt), ionomer solution (PA4IM‐x polymers as ionomer, 5 wt.% in DMSO), deionized water, and isopropanol alcohol (IPA) were mixed in a glass vial and then dispersed in an ultrasonic bath in an ice bath for 1 h. Subsequently, the well‐dispersed catalyst ink was sprayed evenly on gas diffusion layers (GDLs, Sigracet 36BB) using a hand‐held spray gun. The catalyst loading at both the anode and cathode was controlled in the range of 0.10∼1.0 mg_Pt_ cm^−2^, determined by a gravimetric method. The ionomer content was fixed at 10 wt.% in both electrodes.

#### 4.9.2 Membrane Electrode Assemblies (MEA)

Terphenyl‐based polymers are widely employed in membrane applications due to their rigid aromatic backbone, which imparts excellent thermal, chemical, and mechanical stability [33]. Among the imidazole‐based polymers, PT4IM‐100 exhibits appropriate PA uptake (Table S2). Therefore, PA‐doped PT4IM‐100 was selected as the membrane in fuel cell testing to ensure consistency and a balanced performance in this study. Specifically, the PT4IM‐100 membrane was immersed in 85% H_3_PO_4_ at 80°C until the weight of the membranes was not change (∼72 h). The thickness of all the PA‐doped membranes is about 55 ± 5 µm. MEA with an effective size of 2.8 × 2.8 cm (7.84 cm^2^) was prepared by using two GDEs prepared earlier and sandwiched with a membrane. Then the MEA was obtained after being hot‐pressed at 100°C for 5 min to eliminate the effect of poor contact.

### 4.10 Fuel Cell Testing

Fuel cell performance was evaluated using a test station (CNL energy, Gimpo, Gyeonggi‐do, Korea) to measure the polarization and power density curves. All the cells were tested at 140°C–220°C without any external humidification and backpressure. The MEAs were activated at a constant voltage of 0.2 A/cm^2^ until the current of the cell became stable. The flow rate of dry H_2_ and O_2_ supplied to the anode and cathode was 166 and 670 sccm, respectively. The oxygen electrode is highly sensitive to the oxygen concentration [52], therefore an optimal stoichiometric ratio is required to balance oxygen availability and mass transport resistance. Based on the results (Figure S13), a gas stoichiometry of 1:4 was identified as an efficient and practical operating condition and was thus adopted for subsequent measurements. Importantly, all ionomer performances reported in this work were evaluated under sufficiently high oxygen supply, ensuring that the observed differences primarily reflect the intrinsic properties of the ionomer rather than limitations associated with reactant transport. The stabilities of membranes at 160°C were evaluated through an contant current density, including 0.2 A cm^−2^ under the following conditions: H_2_ (200 sccm)/O_2_ (200 sccm) without backpressure or external humidification.

Electrochemical impedance spectra (EIS) were recorded with an instrument (VSP and VMP3 Booster, Bio‐LogicSAS, Grenoble, France) at 0.20 A/cm^2^ in the frequency range from 1 MHz to 1 Hz. Cyclic voltammetry (CV) was measured at 160°C with an H_2_ flow of 200 sccm and a N_2_ flow of 300 sccm to the anode and cathode, respectively. The CVs were obtained between 0.02 V and 1.20 V at a scan rate of 50 mV/s after purging dry N_2_ for 30 min. Electrochemical surface area (ECSA) was calculated based on the hydrogen desorption peak [55].

(6) $$\begin{array}{ccc} & & ECSA\left(cm^{2}mg_{Pt}^{-1}\right)\\ & & =\frac{Q\left(mA\cdot cm^{-2}\cdot V\right)}{0.21\times 10^{-3}\left(C\cdot cm_{Pt}^{-2}\right)\times S\left(mV\cdot s^{-1}\right)\times m\left(mg\cdot cm^{-2}\right)} \end{array}$$

where Q (mA/cm^2^ V) is the integral of the hydrogen desorption peak in the CV curve, *S* (mV/s) is the scan rate, and *m* (mg/cm^2^) is Pt loading in GDE. The coefficient of hydrogen absorbed by Pt is 0.21 × 10^−3^ C/cm_Pt_^2^.

## Author Contributions

**Ge Chao**: conceptualization, methodology, data curation, investigation, formal analysis, visualization, writing – original draft. **Hyeon Keun Cho**: methodology, investigation, data curation, formal analysis. **Chang Yeon Hyun**: methodology, data curation, investigation. **Shirong Li**: methodology, software, data curation. **Jong Geun Seong**: investigation, data curation. **So Young Lee**: methodology, data curation, funding acquisition, project administration. **Nanwen Li**: conceptualization, methodology, investigation. **Young Moo Lee**: conceptualization, writing – review and editing, writing – original draft, supervision, funding acquisition, resources, project administration.

## Conflicts of Interest

The authors declare no conflicts of interest.

## Supporting information




**Supporting File**: advs75891‐sup‐0001‐SuppMat.docx.

## Data Availability

The data that support the findings of this study are available from the corresponding author upon reasonable request.
