# Supplementary material for: High Free‐Volume Imidazole‐Based Ionomers for High‐Temperature Proton Exchange Membrane Fuel Cells
Source: Adv Sci (Weinh). 2026 Jun 1;13(48):e75891. doi: 10.1002/advs.75891 (PMC13336070; doi:10.1002/advs.75891)
Supplement: Supplementary file 1 [file ADVS-13-e75891-s001.docx]

Supporting Information

High Free-volume Imidazole-based Ionomers for High-temperature Proton Exchange Membrane Fuel Cells

Ge Chao^1^ **|** Hyeon Keun Cho^1^ **|** Chang Yeon Hyun^1^ | Shirong Li^1^ **|** Jong Geun Seong^2,3^ **|** So Young Lee^2^ **|** Nanwen Li^4^ **|** Young Moo Lee^1^

^1^Department of Energy Engineering, College of Engineering, Hanyang University, Seoul 04763, Republic of Korea
^2^Center for Hydrogen and Fuel Cells, Korea Institute of Science and Technology (KIST), 02792, Seoul, Republic of Korea

^3^Division of Energy & Environment Technology, KIST School, University of Science and Technology (UST), Seoul, 02792, Republic of Korea
^4^Faculty of Materials Science and Energy Engineering, Shenzhen University of Advanced Technology, Shenzhen, 518107, China

Ge Chao and Hyeon Keun Cho contributed equally to this work.

**Correspondence**: So Young Lee ([sylee5406@kist.re.kr](mailto:sylee5406@kist.re.kr) ), Nanwen Li ([linanwen@suat-sz.edu.cn](mailto:linanwen@suat-sz.edu.cn)) Young Moo Lee ([ymlee@hanyang.ac.kr](mailto:ymlee@hanyang.ac.kr))

**Keywords**: ionomer **|** poly(aryl imidazole) **|** phosphoric acid doping **|** backbone engineering **|** high temperature proton exchange membrane

**Table of Contents**

[**Scheme S1** | Schematic diagram of the synthesis route of the monomer.	3](#_Toc228280770)

[**Scheme S2** | Schematic diagram of the synthesis route of the PA4IM-100 polymers.	3](#_Toc228280771)

[**Scheme S3** | Chemical structures of the imidazole-based polymers. Polymers in the right were designed to possess the IEC of 2.44 meq g^-1^.	3](#_Toc228280772)

[**Scheme S4** | Schematic diagram of the synthesis route of the PA4IM-x polymers.	4](#_Toc228280773)

[**Figure S1** | ^1^H NMR spectra of TTSBD polymer using DMSO-*d6* as solvent and Dm-TTSBD polymer using CDCl_3_ as solvent, which show a one-to-one correspondence with their characteristic peaks, confirming the successful synthesis.	4](#_Toc228280774)

[**Figure S2** | ^1^H NMR spectra of PA4IM-100 polymers using DMSO-*d6* as solvent.	5](#_Toc228280775)

[**Figure S4** | Thermogravimetric analysis (TGA) trace of PA4IM-x ionomers under different atmospheres. (a) TGA curves recorded under N₂. (b) TGA curves recorded under air.	6](#_Toc228280776)

[**Figure S5** | Dynamic mechanical analysis of imidazole-based membranes under an N_2_ atmosphere from 100 to 400 °C. (a) PT, (b) PB, (c) PF and (d) PS membrane.	6](#_Toc228280777)

[**Figure S6** | Polymer structure and their three-dimensional molecular model: (a) PT, (b) PB, (c) PF and (d) PS.	7](#_Toc228280778)

[**Figure S7** | SEM images of surface morphology of the catalyst layers prepared with different ionomers: (a) PT, (b) PB, (c) PF and (d) PS.	8](#_Toc228280779)

[**Figure S8** | Polarization curves and EIS data, respectively, of fuel cells based on PA4IM-x ionomers and PT4IM PEM (55±5 μm) at (a, b) 140 °C, (c, d) 160 °C (e, f) 180 °C, (g, h) 200 °C and (i, j) 220 °C.	9](#_Toc228280780)

[**Figure S9** | Polarization curves and EIS data, respectively, of fuel cells based on different PA4IM-x ionomers and PT4IM PEM (55±5 μm) at different temperature from 140 °C to 220 °C, (a, b) PT, (c, d) PB, (e, f) PF and (g, h) PS.	10](#_Toc228280781)

[**Figure S10** | Polarization curves and EIS data, respectively, of fuel cells based on different PF ionomer and PT4IM PEM (55±5 μm) at different temperature from 140 °C to 220 °C with different Pt loading, (a, b) 160 °C with Pt loading from 0.1 to 1.0 mg_Pt_ cm^-2^, (c, d) 0.5 mg_Pt_ cm^-2^, (e, f) 0.7 mg_Pt_ cm^-2^ and (g, h) 1.0 mg_Pt_ cm^-2^.	11](#_Toc228280782)

[**Figure S11** | Cross-sectional images of the catalyst layers prepared with varied Pt loading using PF ionomer. (a) 0.1 mg_Pt_ cm^-2^, (b) 0.3 mg_Pt_ cm^-2^, (c) 0.5 mg_Pt_ cm^-2^, (d) 0.7 mg_Pt_ cm^-2^ and (e) 1.0 mg_Pt_ cm^-2^.	12](#_Toc228280783)

[**Figure S12** | **(a)** Polarization curves of fuel cell and **(b)** EIS data based on PF ionomer and PA-doped PT4IM-100 PEM (55±5 μm) measured at 220 °C in H_2_/O_2_ or H_2_/air condition.	12](#_Toc228280784)

[**Table S1** | The polymerization conditions of different polymers.	13](#_Toc228280785)

[**Table S2** | IEC and PA doping level of polymer membranes at 80 °C soaked with 85 % PA for 2-5 d	14](#_Toc228280786)

[**Table S3** | IEC and ADL, PA doping amount and swelling ratio of ionomers at 80 °C for 1-3 days.	14](#_Toc228280787)

[**Table S4** | The performance comparison of the H_2_/O_2_(air) fuel cell using imidazole-based polymer as PEM or different polymer as ionomer.	15](#_Toc228280788)

[**Table S5** | The performance comparison of the H_2_/O_2_(air) fuel cell using other types of polymer materials as membranes and copolymers.	18](#_Toc228280789)

# **Scheme S1** | Schematic diagram of the synthesis route of the monomer.

# **Scheme S2** | Schematic diagram of the synthesis route of the PA4IM-100 polymers.

# **Scheme S3** | Chemical structures of the imidazole-based polymers. Polymers in the right were designed to possess the IEC of 2.44 meq g^-1^.

# **Scheme S4** | Schematic diagram of the synthesis route of the PA4IM-x polymers.

# **Figure S1** | ^1^H NMR spectra of TTSBD polymer using DMSO-*d6* as solvent and Dm-TTSBD polymer using CDCl_3_ as solvent, which show a one-to-one correspondence with their characteristic peaks, confirming the successful synthesis.

# **Figure S2** | ^1^H NMR spectra of PA4IM-100 polymers using DMSO-*d6* as solvent.

**Figure S3** | gel permeation chromatography (GPC) curves of PA4IM-x polymers.

# **Figure S4** | Thermogravimetric analysis (TGA) trace of PA4IM-x ionomers under different atmospheres. (a) TGA curves recorded under N₂. (b) TGA curves recorded under air.

# **Figure S5** | Dynamic mechanical analysis of imidazole-based membranes under an N_2_ atmosphere from 100 to 400 °C. (a) PT, (b) PB, (c) PF and (d) PS membrane.

# **Figure S6** | Polymer structure and their three-dimensional molecular model: (a) PT, (b) PB, (c) PF and (d) PS.

# **Figure S7** | SEM images of surface morphology of the catalyst layers prepared with different ionomers: (a) PT, (b) PB, (c) PF and (d) PS.

# **Figure S8** | Polarization curves and EIS data, respectively, of fuel cells based on PA4IM-x ionomers and PT4IM PEM (55±5 μm) at (a, b) 140 °C, (c, d) 160 °C (e, f) 180 °C, (g, h) 200 °C and (i, j) 220 °C.

# **Figure S9** | Polarization curves and EIS data, respectively, of fuel cells based on different PA4IM-x ionomers and PT4IM PEM (55±5 μm) at different temperature from 140 °C to 220 °C, (a, b) PT, (c, d) PB, (e, f) PF and (g, h) PS.

# **Figure S10** | Polarization curves and EIS data, respectively, of fuel cells based on different PF ionomer and PT4IM PEM (55±5 μm) at different temperature from 140 °C to 220 °C with different Pt loading, (a, b) 160 °C with Pt loading from 0.1 to 1.0 mg_Pt_ cm^-2^, (c, d) 0.5 mg_Pt_ cm^-2^, (e, f) 0.7 mg_Pt_ cm^-2^ and (g, h) 1.0 mg_Pt_ cm^-2^.

# **Figure S11** | Cross-sectional images of the catalyst layers prepared with varied Pt loading using PF ionomer. (a) 0.1 mg_Pt_ cm^-2^, (b) 0.3 mg_Pt_ cm^-2^, (c) 0.5 mg_Pt_ cm^-2^, (d) 0.7 mg_Pt_ cm^-2^ and (e) 1.0 mg_Pt_ cm^-2^.

# **Figure S12** | **(a)** Polarization curves of fuel cell and **(b)** EIS data based on PF ionomer and PA-doped PT4IM-100 PEM (55±5 μm) measured at 220 °C in H_2_/O_2_ or H_2_/air condition.

**Figure S13** | Polarization curves of fuel cell based on PTFE ionomer and a PA-doped PT4IM-100 PEM (55±5 μm) measured at different oxygen gas stoichiometries.

# **Table S1** | The polymerization conditions of different polymers.

| **Polymers** | ***p*-terphenyl (mol)** | **4IM/TFAP**  **(mol/mol)** | **DCM**  **(mL)** | **MSA**  **(mL)** | **TFA**  **(mL)** | **TFSA**  **(mL)** | **Time**  **(h)** | **η_int_**  **(*d*L g^-1^)** | **Mw**  **(g/mol)** | **Mn**  **(g/mol)** | **Mw/Mn** |
| --- | --- | --- | --- | --- | --- | --- | --- | --- | --- | --- | --- |
| **PT4IM-100** | 10 | 11 / 0 | 15 | 6 | 0 | 6 | 2 | 8.0 | - | - | - |
| **PB4IM-100** | 10 | 11 / 0 | 3 | 0 | 0 | 8 | 1.5 | 2.8 | - | - | - |
| **PF4IM-100** | 10 | 11 / 0 | 4 | 0 | 6 | 2 | 0.5 | 5.1 | - | - | - |
| **PS4IM-100** | 10 | 11 / 0 | 15 | 9 | 0 | 3 | 0.5 | 11.87 | 271000 | 95000 | 2.9 |
| **PT4IM-79** | 10 | 8.7 / 2.3 | 10 | 7 | 0 | 8 | 20 | 4.48 | 46000 | 32000 | 1.5 |
| **PB4IM-64** | 10 | 7.0 / 4.0 | 4 | 0 | 3 | 8 | 30 | 9.10 | 111000 | 56000 | 2.0 |
| **PF4IM-72** | 10 | 7.9 / 3.1 | 4 | 0 | 5 | 6 | 1.5 | 12.20 | 508000 | 84000 | 6.0 |

# **Table S2** | IEC and PA doping level of polymer membranes at 80 °C soaked with 85 % PA for 2-5 d

| **Polymer sample** | **IEC (mequiv/g)** | **PA doping amount (%)** |
| --- | --- | --- |
| PT4IM-100 | 3.24 | ~ 200 |
| PB4IM-100 | 4.30 | ＞ 1000 |
| PF4IM-100 | 3.67 | ＞ 1000 |
| PS4IM-100 | 2.44 | ＞ 1000 |

# **Table S3** | IEC and ADL, PA doping amount and swelling ratio of ionomers at 80 °C for 1-3 days.

| **Samples** |  | **ADL**  **_Tit_**^a)^ | **PA doping amount (%)**^a)^ | **SR (%)**^a)^ |
| --- | --- | --- | --- | --- |
| PT | 1 day | 3.9±0.1 | 169±2 | 19±1 |
|  | 2 day | 3.9±0.4 | 173±5 | 20±1 |
|  | 3 day | 3.9±0.2 | 174±10 | 20±1 |
| PB | 1 day | 3.9±0.1 | 174±5 | 22±1 |
|  | 2 day | 3.9±0.4 | 182±12 | 23±1 |
|  | 3 day | 4.0±0.3 | 188±7 | 24±1 |
| PF | 1 day | 6.3±0.3 | 275±9 | 33±1 |
|  | 2 day | 8.1±0.4 | 324±23 | 37±2 |
|  | 3 day | 9.0±0.6 | 349±19 | 41±2 |
| PS | 1 day | 10.7±0.4 | 553±25 | 53±2 |
|  | 2 day | 12.3±0.8 | 695±25 | 62±3 |
|  | 3 day | 13.4±1.0 | 774±36 | 70±7 |

^a)^ Samples were measured after immersion in 85% PA solution at 80 ℃ for 1, 2, and 3 day; SR denotes swelling ratio.

# **Table S4** | The performance comparison of the H_2_/O_2_(air) fuel cell using imidazole-based polymer as PEM or different polymer as ionomer.

| **PEM samples** | **Ionomer** | **The catalyst loading (mg_Pt_ cm^-2^)** | **H_2_/O_2_ flow rate (L min^-1^)** | **Peak power density (W cm^-2^)** | **Pt-mass specific PPD (W mg_Pt_^-1^)^a^** | **Lifetime (h)** | **Ref.** |
| --- | --- | --- | --- | --- | --- | --- | --- |
| PT4IM | PF | A/C: 0.7/0.7 | A/C: 0.166/0.677 | 0.611  (at 160°C/ without backpressure) | 0.873 | 170 h (0.2 A cm^-2^) 160 °C/0%RH, ~0 μV/h | This work |
| PT4IM | PF | A/C: 0.5/0.5 | A/C: 0.166/0.677 | 0.570  (at 160°C/ without backpressure) | 1.14 | - | This work |
| PT4IM | PF | A/C: 0.3/0.3 | A/C: 0.166/0.677 | 0.417  (at 160°C/ without backpressure) | 1.39 | - | This work |
| PT4IM | PF | A/C: 0.1/0.1 | A/C: 0.166/0.677 | 0.170  (at 160°C/ without backpressure) | 1.70 | - | This work |
| PTIm-a/152.3%PA | PBI | A/C: 0.9/0.7 | A/C: 0.06/0.03 | 0.738  (at 160°C/ without backpressure) | 0.82 |  | [1] |
| P(BF_20%_-TP_80%_-Im)/202%PA | - | A/C: 1.0/1.0 | A/C: 0.05/0.15 | 1.085  (at 180°C/ without backpressure) | 1.085 | 720 h (0.4 A cm^-2^) 160 °C/0%RH, ~0 μV/h | [2] |
| P(TP0.9-DBT0.1-EIm) | PBI | A/C: 0.6/0.6 | A/C: 0.06/0.12 | 0.896  (at 160°C/ without backpressure) | 1.49 | - | [3] |
| P(75%TP-25%DMF-EtIm) | - | A/C: 1.0/1.0 | A/C: 0.05/0.03 | ~0.75  (at 160°C/ without backpressure) | 0.75 | - | [4] |
| Co-90%TP-10%CE-Im/169%PA | - | A/C: 0.6/0.6 | A/C: 0.06/0.12 | 0.901  (at 180°C/ without backpressure) | 1.50 |  | [5] |
| PTP-B-TPI5-IM | - | A/C: 1.0/1.0 | A/C: 0.2/0.2 | 0.809  (at 160°C/without backpressure) | 0.809 | 120 h (0.2 A cm^-2^) 160 °C/0%RH, 0.086 mV/h | [6] |
| PTP-co-PBP2F_15_-IM | PTFE | A/C: 1.0/1.0 | A/C: 0.2/0.2 | 1.019  (at 180°C/without backpressure) | 1.019 | 120 h (0.3 A cm^-2^) 160 °C/0%RH, 0.31 mV/h | [7] |
| PTP-IM | PTFE | A/C: 1.0/1.0 | A/C: 0.2/0.2 | 0.492  (at 180°C/without backpressure) | 0.492 | - | [7] |
| PA-TABPP | PTFE | A/C: 1.0/1.0 | A/C: 0.20/0.40 | 0.760  (at 160°C/without backpressure) | 0.76 | - | [8] |
| PA-TABPF | PTFE | A/C: 1.0/1.0 | A/C: 0.20/0.40 | 0.658  (at 160°C/without backpressure) | 0.658 | 50 h (0.4 A cm^-2^) 160 °C/0%RH | [8] |
| PSBI-IM/PA | PTFE | A/C: 0.6/0.6 | A/C: 0.40/0.40 | 0.615  (at 160°C/without backpressure) | 1.025 | 500 h (0.2/0.4 A cm^-2^) 160 °C/0%RH | [9] |
| PAES-MIm | - | A/C: 0.6/0.6 | A/C: 0.08/0.16 | 0.423  (at 160°C/without backpressure) | 0.705 | - | [10] |
| PVC-19%APIm/2.4PA | - | A/C: 0.6/0.6 | A/C: 0.10/0.20 | 0.172  (at 180°C/without backpressure) | 0.287 | 70 h (0.2 A cm^-2^) 160 °C/0%RH | [11] |
| C-Ad-10/PA | PTFE | A/C: 0.5/0.5 | A/C: 0.20/0.20 | 0.625  (at 160°C/without backpressure) | 1.25 | 200 h (0.2 A cm^-2^) 160 °C/0%RH | [12] |
| 2-CIMPIM | - | A/C: 0.6/0.6 | A/C: 0.30/0.15 | 0.375  (at 160°C/without backpressure) | 0.625 | - | [13] |
| IMPPO-40 | - | A/C: 1.0/1.0 | A/C: 0.10/0.30(Air) | 0.20  (at 160°C/without backpressure) | 0.20 | - | [14] |
| TP-4-IM | PTFE | A/C: 0.5/0.5 | A/C: 0.20/0.20 | 0.528  (at 160°C/without backpressure) | 1.056 | - | [15] |

# **Table S5** | The performance comparison of the H_2_/O_2_(air) fuel cell using other types of polymer materials as membranes and copolymers.

| **PEM samples** | **Ionomer** | **The catalyst loading (mg_Pt_ cm^-2^)** | **H_2_/O_2_ flow rate (L min^-1^)** | **Peak power density (W cm^-2^)** | **Pt-mass specific PPD (W mg_Pt_^-1^)^a^** | **Lifetime (h)** | **Ref.** |
| --- | --- | --- | --- | --- | --- | --- | --- |
| PTG-10 | PTFE | A/C: 1.0/1.0 | A/C: 0.15/0.20 | 1.287  (at 160°C/without backpressure) | 0.92 | 50 h (0.2 A cm^-2^)160 °C/0%RH | [16] |
| POXIH-0.2QA | PTFE | A/C: 1.1/1.1 | A/C: 0.50/0.50 | 1.0  (at 180°C/without backpressure) | 0.91 | 100 h (0.2 A cm^-2^)160 °C/0%RH | [17] |
| PTIP-20 | - | A/C: 1.0/1.0 | A/C: 0.08/0.16 | 0.678  (at 160°C/without backpressure) | 0.678 | 100 h (0.2 A cm^-2^)140 °C/0%RH, 450 μV/h | [18] |
| PTP-10cPIM | PTFE | A/C: 1.0/1.0 | A/C: --/-- | 0.76  (at 180°C/ without backpressure) | 0.76 | 100 h (0.15 A cm^-2^)140 °C/0%RH, 530 μV/h | [19] |
| PVC-19%APIm/2.4PA | - | A/C: 0.6/0.6 | A/C: 0.10/0.20 | 0.172  (at 180°C/ without backpressure) | 0.287 | 70 h (0.2 A cm^-2^)160 °C/0%RH | [11] |
| C-Ad-10/PA |  | A/C: 0.5/0.5 | A/C: 0.20/0.20 | 0.625  (at 160°C/ without backpressure) | 1.25 | 200 h (0.2 A cm^-2^)160 °C/0%Rh | [12] |
| PIB-GTA | PBI | A/C: 0.7/0.7 | A/C: 0.12/0.06 | 0.632  (at 160°C/ without backpressure) | 0.90 | 160 h (0.2 A cm^-2^) 160 °C/0%RH | [20] |
| CL-5%PECH | PBI | A/C: 1.0/1.0 | A/C: 0.06/0.03 | 0.302  (at 160°C/ without backpressure) | 0.302 | - | [21] |
| PBI | SPX | A/C: 0.5/0.5 | A/C: 0.5/0.5 | 0.27  (at 160°C/ without backpressure) | 0.54 | - | [22] |
| PBI | PA-SPX | A/C: 0.5/0.5 | A/C: 0.5/0.5 | 0.73  (at 160°C/ without backpressure) | 1.46 | - | [22] |
| OPBI | PSPF-P | A/C: 1.0/1.0 | A/C: 0.25/0.5 | 0.48  (at 160°C/ without backpressure) | 0.48 | - | [23] |
| PBI | A:PTFE(20%)  C:PWN100(10%) | A/C: 0.5/0.7 | A/C: -/- | 1.06  (at 160°C/ without backpressure) | 1.51 | 70 h (0.15 A cm^-2^) 160 °C/0%RH | [24] |
| mPBI | F6PBI | A/C: 0.5/0.5 | A/C: 0.2/0.2 | 0.705  (at 160°C/ without backpressure) | 1.41 | 800 h (0.3 A cm^-2^) 160 °C/0%RH | [25] |
| mPBI | Trip-PBI | A/C: 0.5/0.5 | A/C: 0.2/0.2 | 0.700  (at 160°C/ without backpressure) | 1.4 | 100 h (0.3 A cm^-2^) 160 °C/0%RH | [26] |

**References**

[1]	T. Mu, L. L. Wang, Q. Wang, et al., "High-performance imidazole-containing polymers for applications in high temperature polymer electrolyte membrane fuel cells," *J. Energy Chem.* 98 (2024): 512-523. <https://doi.org/10.1016/j.jechem.2024.07.017>.

[2]	L. L. Wang, S. Celenk, Q. Wang, et al., "Long-Durability Poly(dibenzofuran-co-terphenyl N-methylimidazole) Copolymer Membranes for High-Temperature Polymer Electrolyte Membrane Fuel Cells," *Macromolecules* 58 (2025): 5344-5355. <https://doi.org/10.1021/acs.macromol.5c00024>.

[3]	J. N. Su, Q. Liao, L. Li, et al., "π-Conjugated Dibenzothiophene-Containing Poly(Arylene Ethylimidazole) Copolymers for HT-PEMFCs," *J. Membr. Sci.* 736 (2025): 124627. <https://doi.org/10.1016/j.memsci.2025.124627>.

[4]	L. Li, Q. Liao, R. X. Lv, et al., "High-Performance Ether-Free Poly(terphenyl-co-9,9-dimethylfluorene-ethylimidazole) Membranes for High Temperature Proton Exchange Membrane Fuel Cells," *ACS Sustainable Chem. Eng.* 13 (2025): 13656-13666. <https://doi.org/10.1021/acssuschemeng.5c06214>.

[5]	L. L. Wang, Q. Wang, P. R. Lv, et al., "Synthesis of poly(terphenyl-co-dibenzo-18-crown-6 methylimidazole) copolymers for high-performance high temperature polymer electrolyte membrane fuel cells," *J. Membr. Sci.* 714 (2025): 123416. <https://doi.org/10.1016/j.memsci.2024.123416>.

[6]	T. Q. Yang, X. F. Ye, Y. Tang, et al., "Proton Donor-Acceptor Dual-Function Imidazole Cores Enable High-Performance Branched High-Temperature Proton Exchange Membranes," *J. Membr. Sci.* 741 (2026): 125042. <https://doi.org/10.1016/j.memsci.2025.125042>.

[7]	T. Q. Yang, W. Y. Jin, Y. Tang, et al., "Fluorine-induced microphase separation & acid redistribution: A dual-regulation strategy toward high temperature proton exchange membranes with broad-temperature adaptability and exceptional durability," *J. Membr. Sci.* 733 (2025): 124325. <https://doi.org/10.1016/j.memsci.2025.124325>.

[8]	X. Y. Hu, Y. Ao, Y. T. Gao, et al., "Facile preparation of triazole-functionalized poly(arylene perfluorophenyl) high temperature proton exchange membranes via para-fluoro-thiol click reaction with high radical resistance," *J. Membr. Sci.* 687 (2023): 122102. <https://doi.org/10.1016/j.memsci.2023.122102>.

[9]	B. H. Liu, Q. Liu, Y. Pang, et al., "Antioxidant Sulfide-Linked Polymer Membrane with Inherent Microporosity Enables Fuel Cells to Achieve Outstanding Power Density and Durability Over a Wide Temperature Range," *Adv. Funct. Mater.* 35 (2025): 2408291. <https://doi.org/10.1002/adfm.202408291>.

[10]	J. Wang, Y. Dai, R. Y. Wan, et al., "Grafting free radical scavengers onto polyarylethersulfone backbones for superior chemical stability of high temperature polymer membrane electrolytes," *Chem. Eng. J.* 413 (2021): 127541. <https://doi.org/10.1016/j.cej.2020.127541>.

[11]	R. H. Liu, Y. Dai, J. Y. Li, et al., "1-(3-Aminopropyl)imidazole functionalized poly(vinyl chloride) for high temperature proton exchange membrane fuel cell applications," *J. Membr. Sci.* 620 (2021): 118873. <https://doi.org/10.1016/j.memsci.2020.118873>.

[12]	L. Huang, J. Y. Guan, X. Sun, et al., "High Free Volume Crosslinked Membranes Constructed by Stereocrosslinker for High-temperature Proton-exchange Membrane Fuel Cells," *J. Membr. Sci.* 709 (2024): 123100. <https://doi.org/10.1016/j.memsci.2024.123100>.

[13]	S. Zhou, J. Guan, Z. Li, et al., "Alkaline Polymers of Intrinsic Microporosity: high-Conduction and Low-Loss Anhydrous Proton Exchange Membranes for Energy Conversion," *J. Mater. Chem. A* 9 (2021): 3925-3930. <https://doi.org/10.1039/d0ta12100b>.

[14]	J. Jang, D. Kim, C. Pak, et al., "Acid-Doping Induced Phase Separation for Shaping Phase Morphology and Enhancing Performance of Polymer Electrolyte Membranes," *ACS Appl. Energy Mater.* 7 (2024): 7964-7973. <https://doi.org/10.1021/acsaem.4c01547>.

[15]	G. Chao, H. Y. Tang, R. Y. Li, et al., "Nitrogen Heterocyclic Polymers with Different Acidophilic Properties as Proton Exchange Membranes and Minders for High-Temperature Fuel Cells," *J. Membr. Sci.* 692 (2024): 122297. <https://doi.org/10.1016/j.memsci.2023.122297>.

[16]	T. T. Li, B. H. Liu, S. C. Chai, et al., "Highly Conductive Polymer Electrolyte Membranes with Polyoxometalate Hybrid Nanodomains for High-Temperature Fuel Cells," *Nano Lett.* 25 (2025): 11149-11155. <https://doi.org/10.1021/acs.nanolett.5c02701>.

[17]	S. F. Chen, Z. Ma, J. L. Zhang, et al., "Spacer-engineering construction of continuous proton transport networks for cardo poly(biphenyl indole) high-temperature proton exchange membranes," *J. Mater. Chem. A* 12 (2024): 17243-17251. <https://doi.org/10.1039/d4ta02111h>.

[18]	X. F. Hao, Z. Li, M. Xiao, et al., "Intermolecular Acid-Base-Pairs Containing Poly (p-Terphenyl-co-Isatin Piperidinium) for High Temperature Proton Exchange Membrane Fuel Cells," *Energy Environ. Mater.* 7 (2024): e12621. <https://doi.org/10.1002/eem2.12621>.

[19]	B. H. Liu, Y. T. Duan, T. T. Li, et al., "Poly(triphenylene-piperidine) membranes reinforced by carboxy intrinsic microporous polymers towards high output power at low phosphoric acid levels for HT-PEMFC," *J. Membr. Sci.* 692 (2024): 122273. <https://doi.org/10.1016/j.memsci.2023.122273>.

[20]	M. Zhang, M. Liu, T. Wang, et al., "Synthesis of base-acid pair based poly(isatin arylene) membranes for HT-PEMFC applications," *J. Membr. Sci.* 712 (2024): 123202. <https://doi.org/https://doi.org/10.1016/j.memsci.2024.123202>.

[21]	L. Li, L. Guo, L. Wang, et al., "Covalently crosslinked poly(biphenyl dimethylamino benzene) membranes for high temperature proton exchange membrane fuel cells," *Polymer* 300 (2024): 126992. <https://doi.org/https://doi.org/10.1016/j.polymer.2024.126992>.

[22]	S. Yang, H. Li, W. H. Zou, et al., "Sulfonated Microporous Polyxanthene Binder for High-Temperature Hydrogen Fuel Cells," *JACS Au* 4 (2024): 3277-3283. <https://doi.org/10.1021/jacsau.4c00565>.

[23]	X. Sun, J. Guan, X. Wang, et al., "Phosphonated Ionomers of Intrinsic Microporosity with Partially Ordered Structure for High-Temperature Proton Exchange Membrane Fuel Cells," *ACS Cent. Sci.* 9 (2023): 733-741. <https://doi.org/10.1021/acscentsci.3c00146>.

[24]	Y. Y. Hu, Z. X. Xia, C. R. Yang, et al., "Phosphonated ionomer modulates electrochemical interfaces in high temperature polymer electrolyte membrane fuel cells," *J. Energy Chem.* 103 (2025): 850-857. <https://doi.org/10.1016/j.jechem.2024.11.055>.

[25]	G. Chao, H. Tang, N. Li, et al., "Fluorinated Polybenzimidazole as Binders for High-Temperature Proton Exchange Membrane Fuel Cells," *J. Power Sources* 556 (2023): 232473. <https://doi.org/10.1016/j.jpowsour.2022.232473>.

[26]	Q. Ju, H. Tang, H. Dong, et al., "Excellent High-Temperature Proton Exchange Membrane Fuel Cell Derived from A Triptycene-based Polybenzimidazole with Low N-H Density and High Phosphate Tolerance," *J. Membr. Sci.* 683 (2023): 121788. <https://doi.org/10.1016/j.memsci.2023.121788>.
